# Draft genome of the European medicinal leech *Hirudo medicinalis* (Annelida, Clitellata, Hirudiniformes) with emphasis on anticoagulants

**DOI:** 10.1038/s41598-020-66749-5

**Published:** 2020-06-18

**Authors:** Sebastian Kvist, Alejandro Manzano-Marín, Danielle de Carle, Peter Trontelj, Mark E. Siddall

**Affiliations:** 1 Department of Natural History, Royal Ontario Museum, 100 Queen’s Park, Toronto, ON M5S 2C6 Canada; 2 Department of Ecology and Evolutionary Biology, University of Toronto, 25 Willcocks Street, Toronto, ON M5S 2B4 Canada; 3 Centre for Microbiology and Environmental Systems Science, University of Vienna, 1090 Vienna, Austria; 4 Department of Biology, Biotechnical Faculty, University of Ljubljana, Jamnikarjeva 101, 1000 Ljubljana, Slovenia; 5 Division of invertebrate Zoology, American Museum of Natural History, 79th Street @ Central Park West, New York, NY 10025 USA

**Keywords:** Evolutionary biology, Genome, Sequencing, Eukaryote, Phylogenetics

## Abstract

The European medicinal leech has been used for medicinal purposes for millennia, and continues to be used today in modern hospital settings. Its utility is granted by the extremely potent anticoagulation factors that the leech secretes into the incision wound during feeding and, although a handful of studies have targeted certain anticoagulants, the full range of anticoagulation factors expressed by this species remains unknown. Here, we present the first draft genome of the European medicinal leech, *Hirudo medicinalis*, and estimate that we have sequenced between 79–94% of the full genome. Leveraging these data, we searched for anticoagulation factors across the genome of *H. medicinalis*. Following orthology determination through a series of BLAST searches, as well as phylogenetic analyses, we estimate that fully 15 different known anticoagulation factors are utilized by the species, and that 17 other proteins that have been linked to antihemostasis are also present in the genome. We underscore the utility of the draft genome for comparative studies of leeches and discuss our results in an evolutionary context.

## Introduction

Notwithstanding the significance of the European medicinal leech *Hirudo medicinalis* Linnaeus, 1758 as a medical tool from antiquity through modern science-based medicine, it is remarkable that this species’ repertoire of anticoagulants and other bioactive salivary proteins has escaped published in-depth investigation (but see^1^). In order to maintain blood flow in the tissues surrounding the incision wounds during their extended periods of feeding and, importantly, keeping the blood from clotting inside of the crop during the periods of digestion, bloodfeeding leeches secrete a variety of bioactive compounds from their salivary glands. Over 20 different compounds relating to antihemsotasis have been isolated from leech saliva including, for example, direct thrombin inhibitors, factor Xa inhibitors, trypsin inhibitors, endoglucuronidases and antiplatelet proteins^2,3^. Despite its historical significance in medicine, the detailed composition of the anticoagulant profile of *Hirudo medicinalis* Linnaeus, 1758 has escaped scrutiny insofar as modern studies have been concerned primarily with the commercially available species *Hirudo verbana* (Carena, 1820)^4–7^; but see also^1,8^. Whereas *H. medicinalis* was long thought to be the centerpiece for the clinical application of leeches in hospital settings, recent investigations have shown that *Hirudo verbana* is far more commonly employed for this purpose^4^ yet sold under the name *H. medicinalis*; this confusion also prompted the designation of a neotype specimen for *H. medicinalis* in order to ameliorate future taxonomic confusion^9^. The utility of leeches in modern medicine focuses on post-surgical procedures, chiefly following digit replantation or skin grafting surgery^10,11^ wherein the build-up of blood in the extremities and resulting congestion of the veins is relieved by their application. By contrast, medieval medicinal practices, the pinnacle of leech-centric phlebotomy, focused on restoring the balance between bodily  humors to treat or prevent a variety of conditions^12^. The exceedingly frequent use of leeches caused overharvesting of several species, including *Hirudo medicinalis*^13^. Indeed, such was the level of harvesting in the 18^th^ and 19^th^ centuries that the collection of both *H. medicinalis* and *H. verbana* are still protected and governed by the Convention on International Trade in Endangered Species of Wild Flora and Fauna (CITES) (see^14^ for an in-depth treatise of the species).

Exploring the diversity of anticoagulant-related proteins and framing these in an evolutionary context in terms of presence or absence of orthologues across the phylogeny of leeches would help evince the general evolution of these proteins. Members of several leech families have been investigated at the transcriptome level for compositions of the anticoagulant repertoires, including members of Piscicolidae, Glossiphoniidae, Macrobdellidae, Haemadipsidae, Praobdellidae and Hirudinidae^3,8,15–24^. Genome-level evaluations within a species like *H. medicinalis* would allow insights into the diversity of bioactive proteins available. Exploring the copy number of anticoagulant proteins across a draft genome would also provide comparative data for future studies on putative duplication events, co-option of genes, and alternative splicing events. Beyond this, our draft genome should, when coupled with that of the non-bloodfeeding glossiphoniid *Helobdella robusta* (Shankland, Bissen & Weisblat, 1992) (see^25^), aid a larger diversity of studies into leech evolution and allow for comparative analyses between leeches at the genome level.

## Results

### Assembly statistics and BUSCO analysis

The draft genome of *Hirudo medicinalis* ROMIZI 11733 was assembled to 19,929 scaffolds spanning 176.96 Mbps with a median coverage of 146.78×, an N50 score of 50,382 bps, and an L50 score of 772 (full statistics for the draft genome assembly can be found in Supplementary File 1). BUSCO ver. 4.0.5^26^ was run to assess completeness of the genome. The analysis of the predicted proteins vs. the metazoa_odb10 resulted in a completeness score of 94.2% (90.0% complete + 4.2% fragmented).

The assembly size represents 78.67% of the estimated genome size (230 Mbps; http://genomesize.com). A two-pass annotation with MAKER^27^ resulted in 35,166 predicted proteins with 780 splice variants. Using tRNAscan-SE^28^, a total of 429 tRNA genes were predicted, as well as an additional 116 pseudogenes. An additional 535 non-coding RNA genes/motifs were predicted using Infernal^29^, which included 64 rRNA genes and 316 microRNAs. The repetitive content of the genome was estimated at 24.71% (14.43% interspersed and 10.28% simple repeats) by RepeatModeler^30^, with the most abundant unit being unclassified (6.14%). All annotation files have been deposited at 10.5281/zenodo.3555585 (last accessed January 20th 2020). The raw reads, as well as assembled sequences have been deposited in the European Nucleotide Archive (ENA) under the study accession PRJEB35865.

### Leech anticoagulants, copy number and tandem repeats

In total, gene products were found in the *Hirudo medicinalis* genome that showed adequate BLASTp hits (superior to 1E^−5^) against 18 well-characterized leech-derived proteins with functions related to antihemostasis (Table 1). These include eglin C, destabilase I, ghilanten, leech-derived tryptase inhibitor (LDTI), guamerin, cystatin, hirudin, hirudin-like factor 3, ficolin, Kazal-type serine protease inhibitors (serpins), C-type lectin, manillase, bdellin, piguamerin, antistasin, bdellastasin, lefaxin and an unidentified thrombininhibitor. Table 1 shows the top hits from the *H. medicinalis* genome, together with the hits against the three global databases, the copy number of the gene throughout the genome, and the presence or absence of a signal peptide.

**Table 1:** Known, leech-derived antihemostatis-related proteins with high scoring matches in the *Hirudo medicinalis* genome.

Gene ID	Copy number (number of scaffolds)	Local database (e-value)	BLASTp GenBank nr.	BLASTp Swiss-Prot (e-value)	BLASTp Pfam (e-value)	Signal peptide (position)
genemark-SCF_000250-processed-gene-0.16-mRNA-1	3 (1)	Kazal-type serpin (1.0E^−9^)	Hypothetical protein (2.0E^−18^)	—	ADAM (2.0E^−9^)	No
maker-SCF_083185-snap-gene-0.7-mRNA-1	7 (6)	Destabilase I (2.0E^−68^)	Destabilase I (1.0E^−64^)	Lysozyme 1 (3E^−36^)	Destabilase (3.4E^−34^)	No
maker-SCF_089483-snap-gene-0.16-mRNA-1	2 (1)	Guamerin (4.0E^−27^)	Guamerin (6.0E^−22^)	Guamerin (9.5E^−31^)	Antistasin (1.9E^−9^)	Yes (1–26)
snap-masked-SCF_090545-processed-gene-0.8-mRNA-1	1 (1)	Piguamerin (3.0E^−10^)	Guamerin (2.0E^−10^)	Piguamerin (1.7E^−9^)	Antistasin (8.0E^−10^)	No
genemark-SCF_090191-processed-gene-0.18-mRNA-1	2 (1)	C-type lectin (4.0E^−41^)	Ladderlectin-like (3.0E^−17^)	C-type lectin mannose-binding isoform (4.2E^−61^)	Lectin C (2.7E^−17^)	Yes (1–19)
maker-SCF_090707-snap-gene-0.62-mRNA-2	6 (3)	Ficolin (1.0E^−93^)	Microfibril-associated glycoprotein (2.0E^−68^)	Ficolin-1 (5E^−119^)	Fibrinogen C (1.6E^−69^)	Yes (1–23)
genemark-SCF_090790-processed-gene-0.8-mRNA-1	7 (4)	Eglin C (8.0E^−47^)	Eglin C (4.0E^−43^)	Eglin C (7.6E^−46^)	Potato inhibit (6.8E^−18^)	Yes (1–16)
maker-SCF_090848-snap-gene-0.50-mRNA-1	1 (1)	Hirudin (3.0E^−57^)	Hirudin (1.0E^−54^)	Hirudin-3 (2.7E^−44^)	Hirudin (2.4E^−43^)	Yes (1–20)
genemark-SCF_090874-processed-gene-0.2-mRNA-1	1 (1)	Ghilanten (6.0E^−11^)	Hypothetical protein (4.0E^−24^)	Antistasin (1.2E^−24^)	Antistasin (4.3E^−14^)	Yes (1–19)
maker-SCF_090848-snap-gene-0.49-mRNA-1	1 (1)	Hirudin-like factor 3 (HLF3) long variant (2.0E^−11^)	Hirudin-like factor 2 (3.0E^−5^)	—		No
maker-SCF_091175-snap-gene-0.45-mRNA-1	2(2)	Manillase (0)	Hyaluronoglucoronidase (0)	Hyaluronoglucuronidase (0)	Glyco hydro 79n (1.1E^−14^)	Yes (1–22)
maker-SCF_091603-snap-gene-0.93-mRNA-1	1 (1)	Leech-derived tryptase inhibitor (LDTI) C (5.0E^−25^)	Leech-derived tryptase inhibitor (6.0E^−21^)	Leech-derived tryptase inhibitor C (1.9eE^−25^)	Kazal 1 (2.7E^−13^)	No
maker-SCF_091764-snap-gene-1.58-mRNA-1	1 (1)	Thrombin inhibitor (5.0E^−6^)	Hypothetical protein (3.0E^−140^)	Cysteine-rich motor neuron 1 protein (2.3E^−26^)	Antistasin (3.8E^−12^)	*Yes (1–36)
maker-SCF_091868-snap-gene-0.33-mRNA-1	1 (1)	Cystatin (2.0E^−40^)	Cystatin B (1.0E^−36^)	Cystatin-B (7.3E^−27^)	Cystatin (2.4E^−17^)	No
snap-masked-SCF_166172-processed-gene-0.2-mRNA-1	2 (2)	Antistasin (6.0E^−14^)	Antistasin (5.0E^−9^)	Antistasin (3.4E^−16^)	Antistasin (4E^−2^)	Yes (1–20)
maker-SCF_089070-snap-gene-0.23-mRNA-1	4 (3)	Bdellastasin (2.0E^−37^)	Bdellastain (1.0E^−33^)	Bdellastasin (1.1E^−46^)	Antistasin (1.1E^−6^)	Yes (1–25)
genemark-SCF_209471-processed-gene-0.1-mRNA-1	4 (2)	Bdellin (6.0E^−31^)	Bdellin (2.0E^−31^)	Bdellin B-3 (3.4E^−36^)	Kazal 1 (7.2E^−13^)	Yes (1–18)
genemark-SCF_090898-processed-gene-0.7-mRNA-1	3 (2)	Lefaxin (3.0E^−8^)	Neurohemerythrin (7.0E^−82^)	Neurohemerythrin (7.9E^−87^)	Hemerythrin (8.6E^−6^)	No

Reciprocal BLASTs were performed for the proteins that showed high matches against the local database of leech anticoagulants. *Signal peptide detected with Phobius v1.01.

Seven out of the 18 putative anticoagulants occur in a single copy across our data (Table 1); note that there is still a chance that more copies are present in the unsequenced parts of the genome. These are piguamerin, hirudin, ghilanten, hirudin-like factor 3, LDTI, the unidentified thrombin inhibitor and cystatin. The highest copy number (n = 7) was found for eglin C and destabilase; whereas copies for the former seemed scattered across the scaffolds (the seven copies occurred on six different scaffolds), the latter included three copies on the same scaffold and another scaffold with two copies.

Whereas most of the anticoagulants targeted here are not positioned adjacent to each other in our draft genome, the following proteins seem to occur in tandem arrays as two or more copies: C-type lectin (with strong conservation of exon and intron sizes between the copies), guamerin (with only low conservation of exon and intron sizes between the copies), Kazal-type serine protease inhibitor (with only low conservation of exon and intron sizes between the copies) and bdellin (with exon sizes being relatively conserved, but intron sizes differing between the copies). Further, LDTI and fully three tandem copies of bdellin are adjacent to each other on scaffold 209471.

### Other bioactive peptides

In addition to the hits against known, leech-derived anticoagulation factors, fully 1,176 hits against 227 different bioactive compounds isolated from bloodfeeding organisms were recovered in the *H. medicinalis* genome; the function and pathways for most of these are remain unknown and, as such, we will only focus on the non-leech bioactive proteins that have been shown to be involved in anticoagulation. Robust hits (superior to 1E^−5^) were retrieved against 23 different proteins that negatively affect the coagulation cascade. These include a disintegrin and metalloproteinase with a thrombospondin motif (ADAMTS), apyrase, Kunitz-type serine protease inhibitor, fibrinogenase, chrysoptin, bothrojaracin, nitric oxide (vasodilator), agglucetin, snaclec, hemorrhagic metalloproteinase kaouthiagin, batroxstatin, thrombin inhibitor (from the Lone Star tick *Amblyomma americanum*), annexin, tabserin, thrombin inhibitor protein (from *Rhodnius prolixus*), snake venom serine protease, chymotrypsin, brasiliensin, cathepsin B, dipetalogastin, achelase, halyxin and antithrombin-III (from the King cobra *Ophiophagus hannah*). Supplementary File 2 shows the hits for these peptides, together with their reciprocal BLAST hits and signal peptide prediction. Several of the reciprocal BLAST hits were against unannotated (*i.e*., “hypothetical protein” or “uncharacterized protein”) genes in the *Helobdella robusta* genome, such that little information can be deduced regarding the identity of the matches. However, we also evaluated inferior hits (but still superior to 1E^−5^) against well-annotated genes in the three global databases. After evaluation of all available information, only the following protein products could not be robustly inferred to be present in the *H. medicinalis* genome (*i.e*., the remaining protein products are all present): chrysoptin, nitric oxide, thrombin inhibitor (from *Amblyomma americanum*), chymotrypsin, dipelogastin and achelase.

### Sequence similarity and pairwise alignments

**Figure 1:**
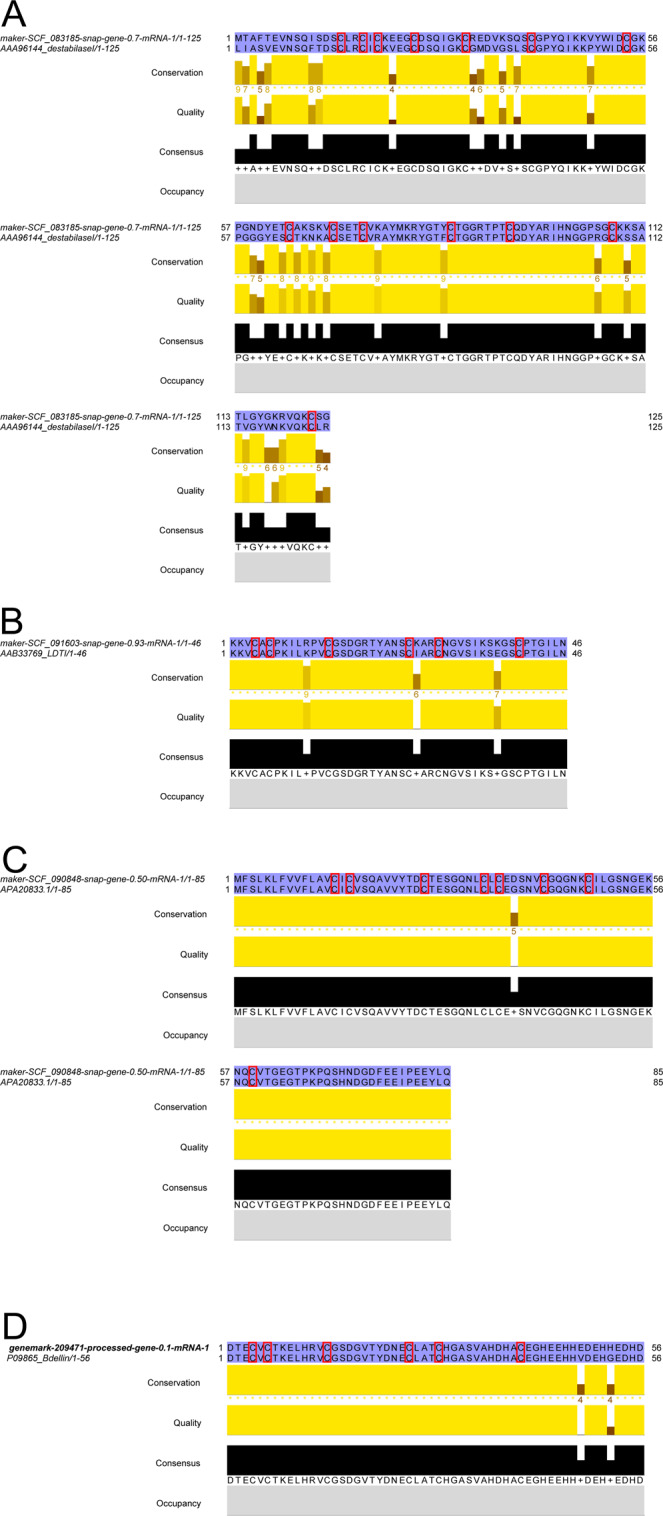
MAFFT-based amino acid alignments of putative anticoagulant orthologues derived from the genome of *Hirudo medicinalis* and the respective top BLASTp hits. (**A**) Putative destabilase I from *H. medicinalis* aligned with the known sequence of the salivary bioactive protein (GenBank accession number AAA96144); (**B**) putative Leech Derived Tryptase Inhibitor (LDTI) from *H. medicinalis* aligned with the known sequence of the salivary bioactive protein (GenBank accession number AAB33769); (**C**) putative hirudin (HV1) from *H. medicinalis* aligned with the known sequence of the salivary bioactive protein (GenBank accession number APA20833); (**D**) putative bdellin from *H. medicinalis* aligned with the known sequence of the salivary bioactive protein (GenBank accession number P09865). Red boxes denote conserved cysteine residues and blue shadings represent conservation of residues between the sequences.

Each of the anticoagulation-related proteins derived from *H. medicinalis* were aligned with their archetypal counterpart, and the alignments are presented in Fig. 1 (for destabilase I, LDTI, hirudin and hirudin-like factor 3, and bdellin) and Supplementary File 3 (for eglin C, ghilanten, guamerin, cystatin, ficolin, the Kazal-type serpin, C-type lectin, manillase, piguamerin, antistasin, bdellastasin and the unidentified thrombininhibitor). Note that the *H. medicinalis* sequence with a hit against lefaxin found a far superior hit against hemerythrin when reciprocally BLASTed and was not further considered an orthologue of lefaxin.

For destabilase I, the newly derived sequence shows 78% similarity in positions with shared amino acids (*i.e*., when gaps are not counted) with the known anticoagulant (GenBank accession number AAA96144^31^), which was also originally derived from *Hirudo medicinalis*. In addition, the positions of all 14 cysteine residues show full conservation, suggesting a similar folding structure between the proteins. No instances of indels were encountered in the alignment (Fig. 1A).

In the amino acid alignment for LDTI, including the new sequence and the archetypal sequence derived from *Hirudo medicinalis* (GenBank accession number AAB33769^32^), the two sequences show 99% sequence similarity and full conservation of the positions of the six cysteine residues. No indels were encountered (Fig. 1B).

The newly sequenced hirudin orthologue shows almost full conservation (sequence similarity 99.9%) when compared to the archetypal sequence (GenBank accession number APA20833^33^), originally derived from *Hirudo verbana*. In addition, positions of the six cysteine residues present in the mature peptide are fully conserved (two cysteines are also conserved in the signal peptide region). No indel events were present in the alignment (Fig. 1C).

The bdellin sequence recovered from the *H. medicinalis* genome shows 99.2% sequence similarity with the archetypal sequence (GenBank accession number P09865^34^) originally derived from *H. medicinalis*, and the positions of all cysteine residues (n = 6) are fully conserved. No indels were present in the alignment (Fig. 1D).

For eglin C, the new sequence shows 99.9% sequence similarity when compared to the archetypal sequence (GenBank accession number 0905140 A^35^). No cysteine residues are present in either sequence and no indels were present (Supplementary File 3A).

The newly acquired ghilanten sequence shows only 32% sequence similarity when compared to the archetypal sequence (GenBank accession number AAB21233^36^) derived from the glossiphoniid leech *Haementeria ghilianii* (de Filippi, 1849). The low affinity between the sequences suggests that these may not be orthologous sequences. Regardless, the new sequence includes 25 cysteine residues in the mature protein and the positions of 17 of these are conserved in the alignment. Indel events were present in both sequences, the largest of which covers 25 residues (insertion in the new sequence or deletion in the archetypal sequence), and only short strings (maximum of n = 3) of homopolymers were identified (Supplementary File 3B).

For guamerin, the new sequence shows 67% sequence similarity with the archetypal protein (GenBank accession number AAD09442^37^), which was originally derived from *Hirudo nipponia* Whitman, 1886. The positions of all nine cysteine residues present in the alignment are fully conserved. No indel events were encountered (Supplementary File 3C).

The new sequence for cystatin shows 57% similarity when compared to its archetypal counterpart (GenBank accession number AAN28679^38^), from the glossiphoniid *Theromyzon tessulatum* (Müller, 1774); the position of the single cysteine residue is conserved between the sequences. No indel events were present in the alignment (Supplementary File 3D).

For ficolin, sequence similarity is 59% between shared amino acid positions between our new sequence and the archetypal sequence (leveraged from the dataset used by Min *et al*.^15^), derived from the North American medicinal leech *Macrobdella decora* (Say, 1824). Two out of the three cysteine residues present in the *M. decora-*derived sequence are also present in the same position in the new sequence. A rather extensive insertion is present in the new sequence (or, alternatively, a deletion event in the archetypal sequence) and covers 27 amino acids residues (Supplementary File 3E).

The newly derived Kazal-type serpin shows only 26% sequence similarity for shared amino acid sites when compared to the *Macrobdella decora*-derived sequence (from the dataset used by Min *et al*.^15^). Out of the 13 cysteine residues present in the “archetypal” sequence, 12 show conserved positions in the new sequence. Short indels are present in both sequences (Supplementary File 3F).

The C-type lectin alignment indicates that 43% sequence similarity exists between the new sequence and the archetypal comparison derived from *M. decora* (see^15^). Thirteen cysteine residues exist in the archetypal sequence and the positions for nine of these are conserved in the newly acquired sequence. Three isolated, short deletions are present in the archetypal sequence (or, alternatively, these represent insertions in the new sequence) (Supplementary File 3G).

For manillase, 83% of the shared amino acid residues are identical between the new sequence and that derived from a US patent application (no. 2006 US 7.049.124 B1P09856) and extracted from the Asian medicinal leech *Hirudinaria manillensis* Lesson, 1842. Notoriously, manillase is completely devoid of cysteine residues, and so is the sequence derived from *Hirudo medicinalis*. An insertion/deletion is present in the middle of the alignment and spans 16 residues (Supplementary File 3H).

For the trypsin inhibitor piguamerin, our newly sequenced gene product shows 46% similarity with the archetypal sequence (GenBank accession number P81499^39^), originally derived from *Hirudo nipponia*. The archetypal sequence includes ten cysteine residues and six of these are in conserved positions in the new sequence. No indels are present in the alignment (Supplementary File 3I).

For antistasin, the sequence derived from our specimen of *H. medicinalis* shows 36% similarity with the archetypal sequence (GenBank accession number P15358^40^) from *Haementeria officinalis*. In addition, the position of 18 out of the 21 cysteines present in the archetypal sequence are conserved between the sequences (Supplementary File 3J).

Our newly derived sequence in the bdellastasin alignment shows almost full conservation (99.9% similarity at shared amino acid sites) when compared to the archetypal variant (GenBank accession number 1C9P^41^), also from *Hirudo medicinalis*. The positions of the 10 cysteines are fully conserved between the sequences (Supplementary File 3K).

The unknown thrombin inhibitor that here serves as the archetypal anticoagulant was originally derived from the piscicolid *Pontobdella macrothela* (Schmarda, 1861) (see^16^) following BLAST-based hits against a putative thrombin inhibitor from the haemadipsid leech *Haemadipsa sylvestris* Blanchard, 1894. Our *Hirudo medicinalis*-derived sequence shows only 28% sequence similarity for shared amino acid sites, yet the positions for seven out of the eight cysteine residues present in the target sequence are fully conserved in the newly acquired sequence. Interestingly, a large insertion of 27 amino acids is present in the middle of the new sequence; alternatively, this is a deletion in the archetypal sequence (Supplementary File 3L).

### Gene trees

For each of the 16 leech-derived putative anticoagulants, we describe the unrooted tree topologies using the terminology proposed by Wilkinson *et al*.^42^, in which a “clan” in an unrooted tree is potentially equivalent to a monophyletic group in a rooted tree and “adjacent group” is equivalent to sister group.

**Figure 2:**
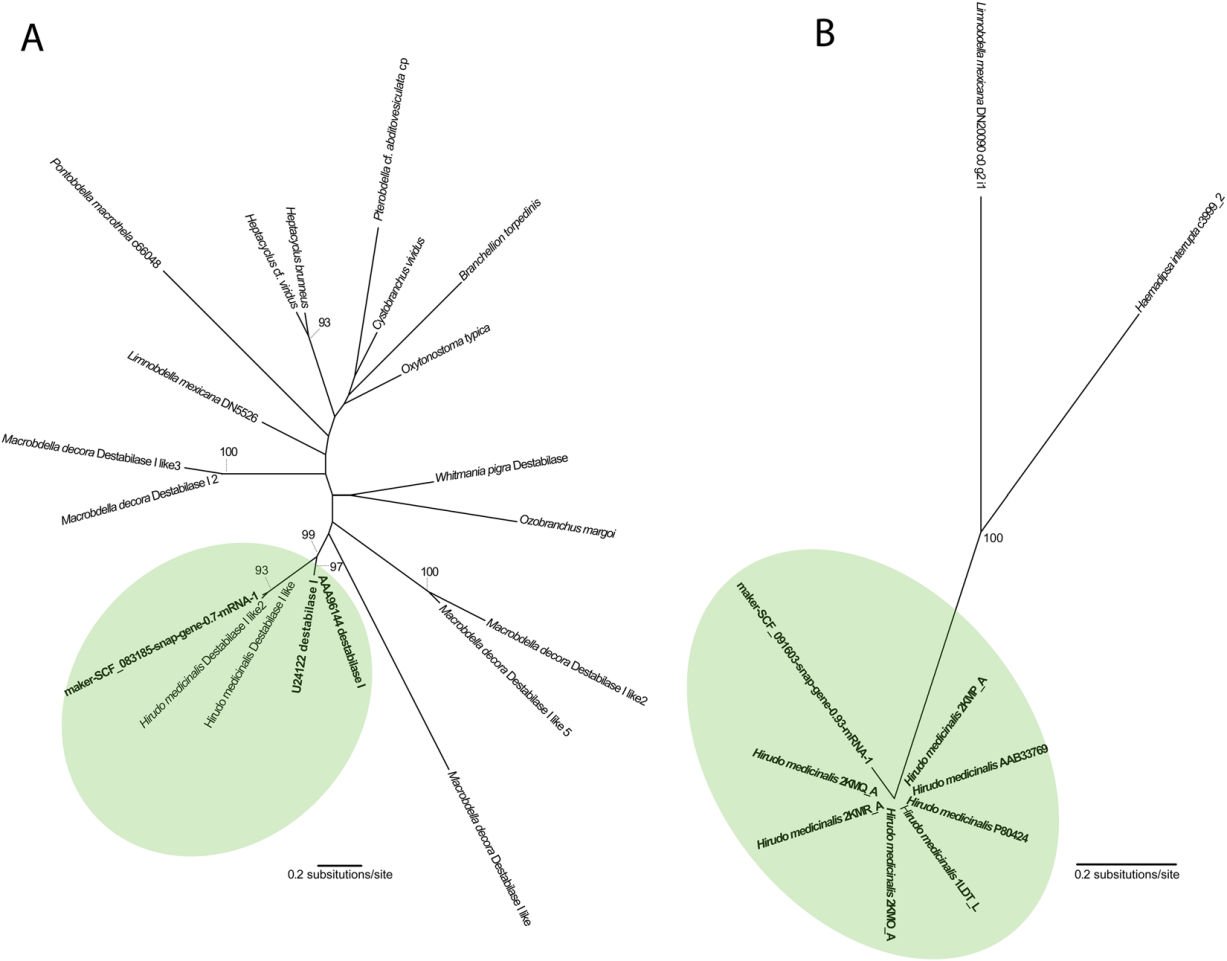
Phylogenetic hypotheses resulting from maximum likelihood analyses of a set of putative orthologues for each anticoagulant or anticoagulant family. (**A**) Destabilase I (ln *L* = −3340.015305); (**B**) LDTI (ln *L* = −640.341632). Green shades indicate the smallest clan that includes both the newly derived sequence and the archetypal variant of the anticoagulant.

In the gene tree for destabilase I (Fig. 2A), the newly acquired sequence forms a clan, albeit with rather low support (likelihood bootstrap support [LBS] = 72%), with the archetypal sequence and several variants retrieved from previous sequencing efforts for *Hirudo medicinalis*^31^.

For LDTI (Fig. 2B), the new sequence forms a clan (LBS = 100%) with all of the archetypal variants of the anticoagulant – note that branch lengths are very short or zero within this cluster of sequences, supporting the notion of orthology between them.

**Figure 3:**
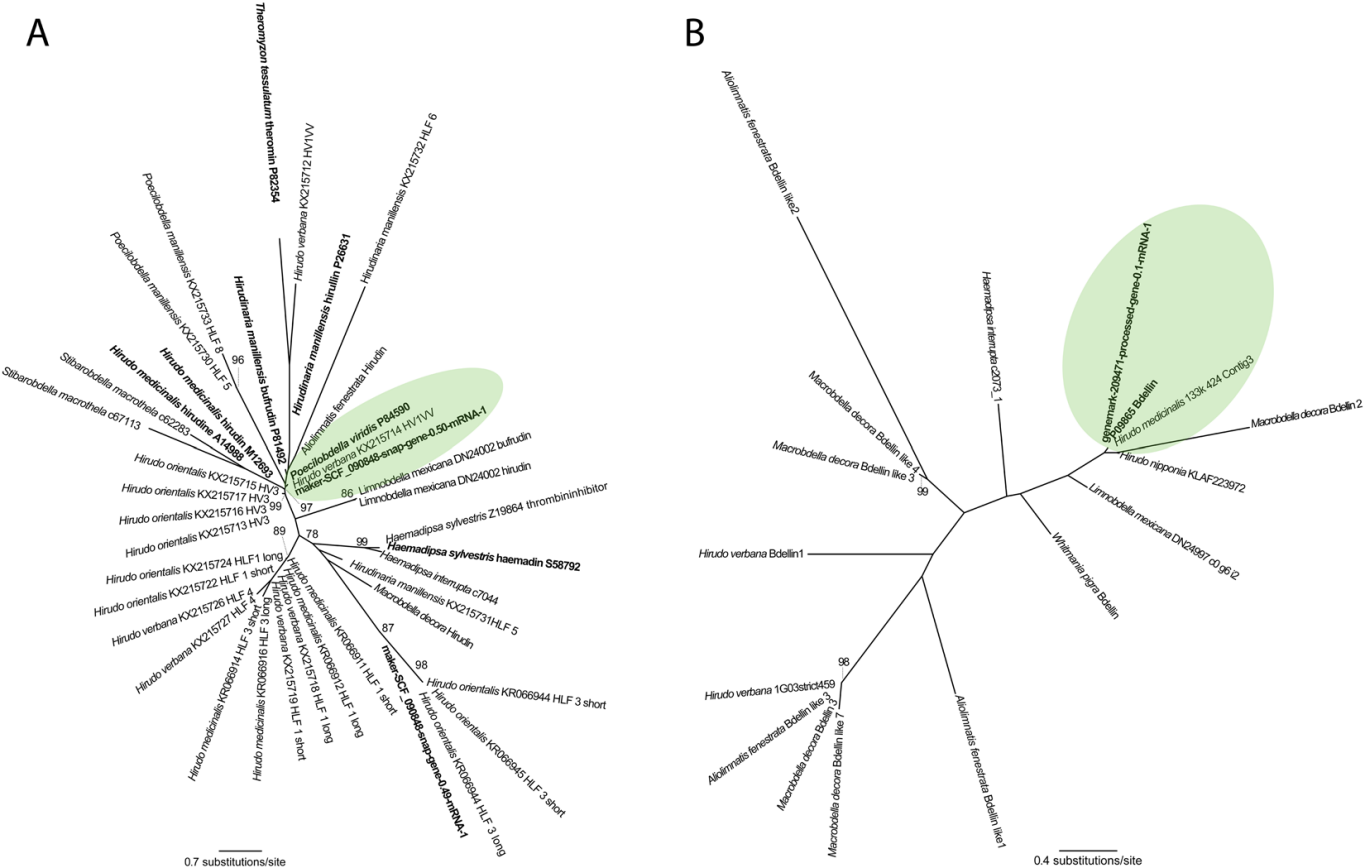
Phylogenetic hypotheses resulting from maximum likelihood analyses of a set of putative orthologues for each anticoagulant or anticoagulant family. (**A**) Hirudin (ln *L* = −4750.252905); (**B**) bdellin (ln *L* = −1771.698797). Green shades indicate the smallest clan that includes both the newly derived sequence and the archetypal variant of the anticoagulant.

Our newly derived hirudin sequence also forms a clan with two archetypal variants of the thrombin inhibitor (LBS = 97%) (Fig. 3A) derived from the hirudinid leech *Poecilobdella viridis* (Blanchard, 1864) and *Hirudo verbana*. Again, the branch lengths are negligible. Moreover, the tree corroborates the BLAST results for hirudin-like factor 3, inasmuch as our newly derived sequence from *H. medicinalis* forms a clan (LBS = 87%) with the archetypal sequence derived from *Hirudo orientalis*.

In the bdellin tree (Fig. 3B), the new sequence forms a clan, albeit with low support, (LBS = 26%) with the archetypal sequence, as well as previously sequenced variants from *Hirudo nipponia, Hirudo medicinalis* and *Macrobdella decora*. The branch length between the new sequence and the archetypal sequence is very short, corroborating the similarity-based homology determination (see above).

For eglin C, the new sequence forms a clan (LBS < 75%) with the archetypal variant; note that the species-level identity of the leech from which the archetypal sequence was derived is unknown. In addition, the clan includes a sequence from a separate sequencing effort for *Hirudo medicinalis* (unpublished), and branch lengths within this clan are very short or zero (Supplementary File 4A).

In the tree constructed from members of the antistasin-family of anticoagulants (Supplementary File 4B), our “ghilanten” sequence from *H. medicinalis* forms a clan (LBS < 75%) with three other sequences from *Heptacyclus* cf. *viridus, Placobdella kwetlumye and Pontobdella macrothela*. This clan is the adjacent group to a clan that includes the archetypal sequence for therostasin, as well as several variants of this gene from various leech species. By contrast, the archetypal sequence for ghilanten forms a clan (with very short branch length) with the archetypal sequence of antistasin, in a far removed section of the unrooted tree. As such, the identity of the newly derived ghilanten sequence is still debatable, but it seems reasonable to suggest that it belongs to therostasin, rather than ghilanten. When compared to therostasin directly (data not shown), the new sequence shows 43%, which is an 11% improvement compared to the alignment with ghilanten (see above). Each of the remaining antistasin-family proteins in the *H. medicinalis* dataset form clans with their respective archetypal anticoagulant. For bdellastasin, piguamerin and guamerin, each of the newly derived sequences nest as the adjacent sequence to the archetypal variants. For antistasin, our sequence places in a larger clan, including sequences from several leech species, as well as the archetypal variants of both ghilanten and antistasin.

For cystatin, only three additional, comparative sequences were available as the basis for the matrix. Despite this paucity of data, the new sequence forms a clan (LBS < 75%) with the archetypal sequence and the branch length is comparable to those for the other terminals (Supplementary File 4C).

Whereas no archetypal, leech-derived sequence is available for ficolin, our new sequence forms a clan together with a variant previously derived (unpublished) from *H. medicinalis* (LBS = 100%), with zero branch length separating the sequences (Supplementary File 4D).

The tree for Kazal-type serine protease inhibitors (Supplementary File 4E) is one of only a few ever constructed for leech sequences and this is manifest in the both the lack of leech-derived archetypal variants and the lack of comparative data (only four sequences make up the matrix). Our new sequence forms a clan (LBS = 51%) with a variant derived from *Haemadipsa interrupta*.

In the C-type lectin tree (Supplementary File 4F), the sequence for the top hit forms an unsupported clan (LBS < 75%) with variants derived from the African medicinal leech *Aliolimnatis fenestrata* and *Hirudo medicinalis*. This clan, in turn, is the adjacent group of the archetypal sequence.

Corroborating the similarity-based orthology determination, our newly derived manillase sequence forms a clan with the archetypal sequence and two other sequences derived from the praobdellid *Limnobdella mexicana* and *Haemadipsa interrupta*. Given this placement and the length of the branch leading to our sequence, there is little doubt that it represents an ortholog of manillase (Supplementary File 4G).

Taken together, the results from the BLAST, alignment and gene tree analyses suggest that each of the following leech-derived protein products are represented in the *H. medicinalis* genome: eglin C, destabilase I, ghilanten, leech-derived tryptase inhibitor (LDTI), guamerin, cystatin, hirudin, ficolin, Kazal-type serine protease inhibitors (serpins), C-type lectin, manillase, bdellin, piguamerin, antistasin, bdellastasin and an unidentified thrombininhibitor.

## Discussion

Our draft genome for *Hirudo medicinalis* and in-depth look at the anticoagulant repertoire in this species provides fodder for comparative studies of anticoagulants in medicinal leech species. High-throughout sequencing employing the 10X Chromium platform allowed us to sequence the genome to an estimated 79–94% completion and evinced 18 separate anticoagulant-related orthologues (or putative orthologues) with BLAST matches against eglin C, destabilase I, ghilanten, leech-derived tryptase inhibitor (LDTI), guamerin, cystatin, hirudin, ficolin, Kazal-type serine protease inhibitors (serpins), C-type lectin, manillase, bdellin, piguamerin, antistasin, bdellastasin and an unidentified thrombininhibitor (Table 1). In addition, our searches found robust hits against 23 other anticoagulant-related proteins from non-leech taxa. Several of the sequences possessed signal peptide regions at the 5’-end, indicating their secretion from the unicellular salivary glands. Although BLAST-based orthology statements have been commonplace in leech anticoagulant studies for almost a decade, without complementary phylogenetic analyses of putative orthologues, the robustness of such statements can be questioned^20^. Following the phylogenetic analyses presented here, it seems likely that 15 of the 16 leech-derived proteins (note that neither lefaxin nor the unidentified thrombin inhibitor was placed in a phylogenetic context due to the uncertainty of their identity and, therefore, comparative data) are indeed orthologous with their top BLAST hits, and the same number for non-leech proteins was 17 out of 23. A large body of literature supports the notion that phylogenetic analyses are needed for accurate orthology and paralogy statements (*e.g*.^43–46^). Given that accurate orthology statements were only made possible via phylogenetic analyses, the present study underscores the importance of incorporating such analyses into any study where orthology is not determined *a priori*. However, the lack of appropriate outgroups for leech anticoagulants presents its own set of issues and certainly exacerbates rapid and accurate orthology determination. In this study, we assumed that *H. medicinalis*-derived proteins that formed a clan (equivalent to a monophyletic group in rooted phylogenies) with its archetypal variant (a protein with demonstrated anticoagulant capabilities) was indeed orthologous to that anticoagulant. The levels of potency in preventing blood clotting for each of our newly sequenced proteins remains to be investigated.

When comparing the list of anticoagulants found in *H. verbana*^6^ to that of the closely related *H. medicinalis*, surprisingly, only bdellin, piguamerin, antistasin, ghilanten, eglin C, manillase and C-type lectin overlap between the two species. The study by Kvist *et al*.^6^ recovered also hirustasin, therostasin and a leukocyte elastase inhibitor, as well as an unnamed plasmin inhibitor. By contrast, that study did not recover hirudin, destabilase I, LDTI, guamerin, bdellastasin, cystatin, ficolin, Kazal-type serpins or the unnamed thrombin inhibitor that were all recovered from *Hirudo medicinalis*. Surely, part of this discrepancy should be attributed to differing sequencing platforms, sequencing depth and imperfect search algorithms but, nevertheless, the data generated here allow for the first comparison between anticoagulants of closely related species of medicinal leeches. At the very least, this sets the stage for in-depth sequencing of the *Hirudo verbana* salivary transcriptome. It should be noted that neither study managed to recover matches against leech antiplatelet proteins (LAPP/saratin), which are commonly encountered in other leech species (see^2,3,6,16,18,20^). Moreover, decorsin, which has so far only been recovered from the North American medicinal leech *Macrobdella decora* was not recovered in either of the studies, perpetuating the notion that this protein may be unique to that species.

The present study adds to the growing body of knowledge of leech anticoagulants and, more generally, the evolution of bloodfeeding in leeches. Relatively few members of Hirudinidae have so far been investigated, with only *Hirudinaria manillensis* and, now, *Hirudo medicinalis* having been queried at the genome level^47^. With the finding of presumably orthologous loci across the leech phylogeny – including antistasin family proteins, manillase and C-type lectin, for example – there is a growing body of evidence suggesting that these are present due to their possession by the common ancestor of all leeches. The present study adds to this and strengthens the case that the ancestral leech was indeed bloodfeeding, or at least possessed proteins that antagonize a normal coagulation cascade.

Moreover, whereas *H. medicinalis* is commonly employed in modern hospital settings to relieve venous congestion, the repertoire of secreted proteins that get injected into the patients has not before been fully known. The presence of hirudin is likely the factor that has afforded *H. medicinalis* its place in both historical and modern medical practices. The present study suggests that the potency of *H. medicinalis* saliva in counteracting the clotting cascade of blood is highly dependent on a larger cocktail of proteins that have several different targets in the cascade.

Whereas the present study focused on anticoagulant diversity and evolution, the creation of a draft genome holds the potential to ignite and inspire comparative studies of leech genomics. With only two genomes previously sequenced for leeches, our new data unlock the potential for investigations into areas that necessitate comparative data, such as associations between genetic variants and phenotypic expression, comparisons of genomic makeup between bloodfeeding and non-bloodfeeding organisms, differences in signaling pathways in the central nervous system, to name a few.

## Material and methods

### Specimen collection and DNA extraction and sequencing

The single specimen of *Hirudo medicinalis* used in the present study- was collected in August 2016 from an unnamed pond close to the town of Kočevje in southeastern Slovenia. The leech was collected by wading into the water and netting swimming leeches. The specimen was identified in the field based on external morphology, following specialized literature^48,49^ and later confirmed by dissections. The leech was relaxed by gradual addition of 95% ethanol to pond water and fixed in 95% ethanol; it was kept at 4 °C until sequencing to avoid DNA degradation by freeze-thawing procedures.

DNA was extracted from full body tissue avoiding the integument- as this tissue has been shown to exacerbate library creation - and digestive tract. That is, the DNA isolate was derived from gonadal, nervous and muscular tissue in a single extract. The tissues were frozen with liquid nitrogen and macerated using a pestle in a RNase-free Eppendorf tube. DNA was extracted using proteinase K digestion overnight, followed by a standard phenol-chloroform extraction protocol. Genomic DNA fragments >30 Kb were size selected using the HighPass protocol on the BluePippin (Sage Science). Sample indexing and partition barcoded libraries were prepared using the Chromium Genome Library and Gel Bead Kit (10X Genomics) according to manufacturer’s protocols. The Chromium Controller was used to combine a library of 10X Genome Gel Beads with high molecular weight template genomic DNA (0.625 ng), a master mix of enzymes and buffer, and partitioning oil to create droplets containing single gel beads and DNA. During the process, genomic DNA was partitioned across approximately 1 million 10X GEMs. An emulsion containing the GEM partitioned reactions was isothermally incubated (for 3 h at 30 °C; for 10 min at 65 °C; held at 4 °C), and barcoded fragments ranging from a few to several hundred base pairs were generated. After amplification, half of the emulsion was collected, and GEMs were broken. Finally, the recovered barcoded DNA was size selected for library preparation. Illumina-specific sample indexing was added to the barcoded fragments to generate libraries according to the manufacturer’s instructions. The barcode sequencing libraries were then quantified by qPCR (KAPA Biosystems Library Quantification Kit for Illumina platforms). Sequencing was conducted across half a lane on the Illumina HiSeq X platform at the New York Genome Center (New York, NY, USA) with 2 × 150 paired-end reads based on the manufacturer’s protocols.

### Genome assembly and annotation

The resulting reads were used as input for Supernova ver. 2.0.1^50^ and a pseudo-haploid representation of the assembly was generated using the subcommand *mkoutput*. The assembly resulted in 21,765 scaffolds, which were then taxonomically assigned using PhymmBL v4.0^51^ in order to remove contaminants. The database for taxonomic assignment consisted of representative reference genomes for Bacteria and Archaea from NCBI, as well as the human reference genome, *Saccharomyces cerevisiae* S288C, and the annelids *Capitella teleta* Blake, Grassle & Eckelbarger, 2009, *Helobdella robusta*, and a preliminary draft genome from the leech *Macrobdella decora* (Say, 1824) (reference genome in progress); the distribution of taxonomic assignments can be found in the Zenodo repository at https://doi.org/10.5281/zenodo.3555585. Two iterative rounds of taxonomic assignment were run, adding the scaffolds assigned to Annelida to the PhymmBL database after each round. A total of 1,836 scaffolds were removed from the *H. medicinalis* draft assembly.

The remaining 19,929 scaffolds underwent a two-step annotation in MAKER v2.31.10^27^ using the transcriptomes of *H. verbana* (GGIQ00000000.1) and *H. medicinalis* (GBRF00000000.1) as EST sets and the predicted proteomes for *Helobdella robusta* and *Capitella teleta* as proteins sets (available from the JGI genome portal [https://genome.jgi.doe.gov/portal/, last accessed January 20^th^ 2020]). Next, the resulting proteins were compared to the SWISS-PROT database (including splice variants) using BLASTp ver. 2.6.0 + ^52^. Additionally, a similarity search was run using InterProScan ver. 5.28-67.0^53^. Gene models with an AED score of 1 were kept only if a significant hit was found. tRNAs were identified using tRNAscan-SE ver. 2.0.0^28^ and ncRNAs and other RNA motifs were searched for using Infernal ver. 1.1.2^29^. Repeated content in the genome was identified using RepeatModeler ver. 1.0.11^30^.

### BLAST and copy number

The full suite of predicted genes in the genome of *H. medicinalis* were BLASTed (using BLASTp) against a local database containing known leech-derived anticoagulants, as well as proteins derived from other bloodfeeding organisms. The top hit for each leech-derived anticoagulant was then reciprocally BLASTed against three separate databases: GenBank non-redundant protein database, SWISS-PROT and Pfam. This was done to ensure that the best hits against leech-derived anticoagulants did not find a better hit against another known protein in the global databases. The sequence was not considered an orthologue of the best hit in the leech-derived anticoagulant database if a better hit was found against a completely unrelated protein in either of the three databases. As well, hits against non-leech-derived proteins that have been shown to affect hemostasis were recovered and scrutinized through the same pipeline. SignalP ver. 5.0^54^ was used to predict potential signal peptides. If not identified, Phobius ver. 1.01^55^ was used as an alternative identifier of a potential signal peptide.

The copy numbers across the genome for each of the hits against well-characterized, leech-derived anticoagulants were estimated by BLASTing the top hit for each protein against the genome. Hits below the cutoff value of 1E^−5^, with a query length of at least 50% of the target and with at least 70% sequence similarity were considered good hits and, therefore, a copy of the query sequence. Because several of the targeted anticoagulation factors share similar amino acid compositions, each of the hits were compared the remaining pool to ensure that hits were not counted twice as separate anticoagulation factors. As an initial check of the positioning of the anticoagulants across the genome, we also assessed whether or not hits are adjacent to each other on the same scaffold.

### Alignments and gene trees

Following the initial BLAST-based homology determination, orthology was determined by sequence similarity and phylogenetic analyses. For each of the hits against well-characterized leech-derived anticoagulant proteins, the top hit was aligned with the amino acid sequence of the known (archetypal) anticoagulant. This was done both to visualize the level of conservation between the sequences and identify any insertions and/or deletions. For this purpose, the two sequences were aligned using the online version of MAFFT ver. 7^56^ using default settings under the “Auto” strategy. JalView ver. 2^57^ was used to visualize the alignments. To maximize regions of homology and to augment visualization of the alignments, leading and lagging gaps were truncated from the alignments, but internal gaps in the sequences (*i.e*., true indels) were maintained. Note that, in some cases, this resulted in the signal peptide region being excised from the sequences (these are still reported in Table 1). From the alignments, percent amino acid conservation, with special emphasis on the conservation of disulphide-bond forming cysteines, was calculated by eye.

Phylogenetic analyses were used to test for corroboration of the BLAST-based orthology determinations. Datasets for each of the hits against well-characterized anticoagulant proteins were constructed using the top BLASTp hit for the protein in our newly acquired dataset, amended by comparative data from the datasets used by Iwama *et al*.^20^ and Khan *et al*.^58^ for each protein. Sequences were again aligned using MAFFT ver. 7 with the same settings as above. The alignments were then subjected to maximum likelihood analyses using RAxML ver. 8.2.12^59^ on the CIPRES Science Gateway Platform^60^. The searches were conducted under a general time reversible model (GTR) and rate heterogeneity was modelled by a gamma distribution using 25 rate categories and an estimated proportion of invariable sites. Support values for nodes were estimated through 1,000 standard bootstrap replicates using default settings. Given the paucity of obvious choices for outgroups for leech anticoagulants, the trees were left unrooted. All alignments, tree files and annotation files have been deposited in Zenodo (10.5281/zenodo.3555585) (last accessed April 6th 2020).

## Supplementary information

Supplementary Information.

Supplementary Information 2.

Supplementary Information 3.

Supplementary Information 4.

## Data Availability

All annotation files have been deposited at 10.5281/zenodo.3555585 (last accessed April 6th 2020). The raw reads, as well as assembled sequences have been deposited in the European Nucleotide Archive (ENA) under the study accession PRJEB35865.
