# Supplementary material for: Draft genome of the European medicinal leech Hirudo medicinalis (Annelida, Clitellata, Hirudiniformes) with emphasis on anticoagulants
Source: Sci Rep. 2020 Jun 18;10:9885. doi: 10.1038/s41598-020-66749-5 (PMC7303139; doi:10.1038/s41598-020-66749-5)
Supplement: Supplementary file 2 — Supplementary Information 2. [file 41598_2020_66749_MOESM2_ESM.docx]

**Draft genome of the European medicinal leech *Hirudo medicinalis* (Annelida, Clitellata, Hirudiniformes) with emphasis on anticoagulants**

Sebastian Kvist^1,2,^*, Alejandro Manzano-Marín^3^, Danielle de Carle^1,2^, Peter Trontelj^4^ & Mark E. Siddall^5^

^1^ Department of Natural History, Royal Ontario Museum, 100 Queen’s Park, Toronto, ON M5S 2C6, Canada

^2^ Department of Ecology and Evolutionary Biology, University of Toronto, 25 Willcocks Street, Toronto, ON M5S 2B4, Canada

^3^ Centre for Microbiology and Environmental Systems Science, University of Vienna, 1090 Vienna, Austria

^4^ Department of Biology, Biotechnical Faculty, University of Ljubljana, Jamnikarjeva 101, 1000 Ljubljana, Slovenia

^5^ Division of invertebrate Zoology, American Museum of Natural History, 79^th^ Street @ Central Park West, New York, NY 10025, USA.

| **Gene ID** | **Local database (e-value)** | **BLASTp GenBank nr.** | **BLASTp Swiss-Prot (e-value)** | **BLASTp Pfam (e-value)** | **Signal peptide (position)** |
| --- | --- | --- | --- | --- | --- |
| maker-SCF_091256-snap-gene-0.27-mRNA-1 | A Disintegrin and metalloproteinase with a thrombospondin motif (1.0E^-113^) | Disintegrin and metalloproteinase domain-containing protein (2.0E^-124^) | Disintegrin and metalloproteinase domain-containing protein (1.7E^-133^) | Reprolysin (M12B) family zimnc metalloprotease (3.5E^-46^) | No |
| genemark-186085-processed-gene-0.0-mRNA-1 | Apyrase (3.0E^-74^) | Hypothetical protein (2.0E^-156^) | Uncharacterized protein (1.9E^-161^) | Apyrase (1.2E^-112^) | No |
| maker-SCF_091206-snap-gene-0.30-mRNA-1 | Kunitz-type serine protease inhibitor (5.0E^-27^) | Kunitz/Bovine pancreatic trypsin inhibitor domain protein (0) | Kunitz BPTI domain containing protein (0) | Spondin-N (4.2E^-64^) | No |
| genemark-SCF_092355-processed-gene-0.4-mRNA-1 | Fibrinogenase precursor (1.0E^-8^) | Chymotrypsinogen A-like (6.0E^-38^) | Peptidase S1domain-containing protein (8.8E^-37^) | Trypsin (1.8E^-22^) | No |
| maker-SCF_089619-snap-gene-0.7-mRNA-1 | Chrysoptin precursor (2.0E^-111^) | Hypothetical protein (3.0E^-152^) | Uncharacterized protein (2.2E^-152^) | 5’-nucleotidase (6.2E^-42^) | No |
| genemark-SCF_000184-processed-gene-0.8-mRNA-1 | Bothrojaracin (5.0E^-7^) | Hypothetical protein (1.0E^-8^) | C-type lectin domain-containing protein (1.2E^-11^) | C-type lectin (2.2E^-3^) | No |
| genemark-SCF_000276-processed-gene-1.12-mRNA-1 | Nitric oxide (4.0E^-67^) | NADH—cytochrome P450 reductase (0) | NADH—cytochrome P450 reductase (0) | FAD Binding domain (4.4E^-62^) | No |
| maker-SCF_092550-snap-gene-0.87-mRNA-1 | Agglucetin (6.0E^-6^) | Hypothetical protein (9.0E^-62^) | C-type lectin domain-containing protein (7.2E^-61^) | Lectin C-type domain (6E^-9^) | No |
| maker-SCF_092217-snap-gene-0.41-mRNA-1 | Snaclec (6.0E^-11^) | Hypothetical protein (3.0E^-172^) | C-type lectin domain-containing protein (4.3E^-177^) | Lectin C-type domain (3.8E^-14^) | No |
| maker-SCF_090899-snap-gene-0.2-mRNA-1 | Hemorrhagic metalloproteinase (9.0E^-10^) | Hypothetical protein (4.0E^-128^) | Peptidase M12B domain-containing protein (1.4E^-29^) | Metallo-peptidase family M12B Reprolysin-like (2.2E^-12^) | No |
| snap_masked-SCF_092067-processed-gene-0.1-mRNA-1 | Batroxstatin (6.0E^-6^) | Hypothetical protein (2E^-17^) | Peptidase M12B domain-containing protein (9.0E^-21^) | Metallo-peptidase family M12 (3.2E^-8^) | No |
| maker-SCF_091086-snap-gene-0.22-mRNA-1 | Thrombin inhibitor [Amblyomma americanum] (1.0E^-26^) | N-cadherin (0) | Uncharacterized protein (0) | Cadherin cytoplasmic region (5.9E^-35^) | No |
| maker-SCF_091966-snap-gene-0.25-mRNA-1 | Annexin (3.0E^-67^) | Hypothetical protein (2.0E^-152^) | Annexin (8.4E^-145^) | Annexin (1.8E^-22^) | No |
| maker-SCF_075215-snap-gene-0.2-mRNA-1 | Tabserin (2.0E^-27^) | Hypothetical protein (1.0E^-99^) | Peptidase S1 domain-containing protein (7.2E^-104^) | Trypsin (4.6E^-53^) | Yes (1-17) |
| genemark-SCF_091726-processed-gene-0.12-mRNA-1 | Thrombin inhibitor [Rhodnius prolixus] (5.0E^-17^) | Hypothetical protein (1.0E^-124^) | Uncharacterized protein (5.8E^-148^) | Kazal-type serine protease inhibitor domain (3.1E^-12^) | Yes (1-26) |
| genemark-SCF_080297-processed-gene-0.0-mRNA-1 | Chymotrypsin (7.0E^-26^) | Hypothetical protein (3.0E^-130^) | Uncharacterized protein (5.1E^-131^) | Trypsin (4.7E^-61^) | Yes (1-23) |
| maker-SCF_096512-snap-gene-0.6-mRNA-1 | Snake venom serine protease (3.0E^-28^) | Hypothetical protein (1.0E^-102^) | Peptidase S1 domain-containing protein (1.1E^-104^) | Trypsin (5.0E^-69^) | No |
| maker-SCF_090072-snap-gene-0.28-mRNA-1 | Brasiliensin (9.0E^-35^) | Four domain proteases inhibitor-like (3.0E^-68^) | Uncharacterized protein (2.3E^-73^) | Kazal-type serine protease inhibitor domain (2.7E^-13^) | No |
| maker-SCF_092825-snap-gene-0.11-mRNA-1 | Cathepsin B (2.0E^-25^) | Hypothetical protein (2.0E^-164^) | Pept C1 domain-containing protein (2.3E^-172^) | Papain family cysteine protease (6.5E^-66^) | Yes (1-22) |
| snap_masked-200626-processed-gene-0.3-mRNA-1 | Dipetalogastin (7.0E^-7^) | Agrin-like protein (1.0E^-11^) | Uncharacterized protein (1.5E^-10^) | Kazal-type serine protease inhibitor domain (2.3E^-6^) | No |
| maker-SCF_091853-snap-gene-1.64-mRNA-1 | Achelase (4.0E^-7^) | Low density lipoprotein receptor-related protein 2 (5.0E^-57^) | Low density lipoprotein receptor-related protein 2 (2.1E^-73^) | Trypsin (1.5E^-30^) | No |
| maker-SCF_089792-snap-gene-0.15-mRNA-1 | Halyxin (6.0E^-10^) | Hypothetical protein (1.0E^-29^) | C-type lectin domain-containing protein (1.5E^-33^) | Lectin C-type domain (3.4E^-12^) | No |
| maker-SCF_090783-snap-gene-0.25-mRNA-1 | Antithrombin III [*Ophiophagus hannah*] (9.0E^-71^) | Alpha-1-antiproteinase 2 (9.0E^-140^) | Uncharacterized protein (1.0E^-142^) | Serine protease inhibitor (4.2E^-116^) | No |
