# Supplementary material for: Draft genome of the European medicinal leech Hirudo medicinalis (Annelida, Clitellata, Hirudiniformes) with emphasis on anticoagulants
Source: Sci Rep. 2020 Jun 18;10:9885. doi: 10.1038/s41598-020-66749-5 (PMC7303139; doi:10.1038/s41598-020-66749-5)
Supplement: Supplementary file 3 — Supplementary Information 3. [file 41598_2020_66749_MOESM3_ESM.pdf]

Draft genome of the European medicinal leech *Hirudo medicinalis* (Annelida, Clitellata, Hirudiniformes) with emphasis on anticoagulants

Sebastian Kvist[1,2,*], Alejandro Manzano-Marín[3], Danielle de Carle[1,2], Peter Trontelj[4] & Mark E. Siddall[5]

[1] Department of Natural History, Royal Ontario Museum, 100 Queen's Park, Toronto, ON M5S 2C6, Canada

[2] Department of Ecology and Evolutionary Biology, University of Toronto, 25 Willcocks Street, Toronto, ON M5S 2B4, Canada

[3] Centre for Microbiology and Environmental Systems Science, University of Vienna, 1090 Vienna, Austria

[4] Department of Biology, Biotechnical Faculty, University of Ljubljana, Jamnikarjeva 101, 1000 Ljubljana, Slovenia

[5] Division of invertebrate Zoology, American Museum of Natural History, 79th Street @ Central Park West, New York, NY 10025, USA.

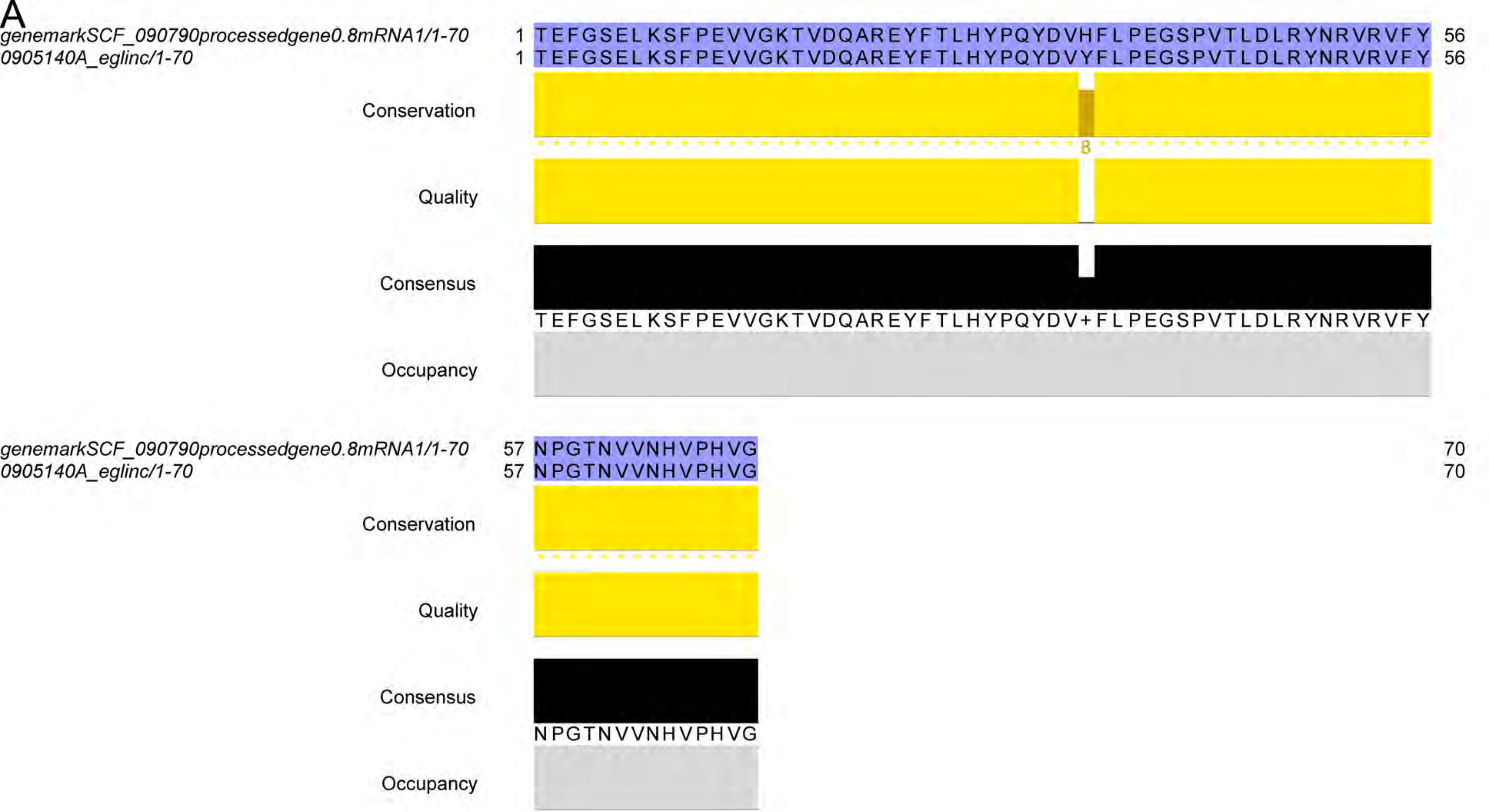
A
genemarkSCF_090790processedgene0.8mRNA1/1-70
0905140A_eglinc/1-70
1 TEFGSELKSFPEVVGKTVDQAREYFTLHYPQYDVHFLPEGSPVTLDLRYNRVRVFY 56
1 TEFGSELKSFPEVVGKTVDQAREYFTLHYPQYDVYFLPEGSPVTLDLRYNRVRVFY 56
Conservation
8
Quality
Consensus
TEFGSELKSFPEVVGKTVDQAREYFTLHYPQYDV+FLPEGSPVTLDLRYNRVRVFY
Occupancy
genemarkSCF_090790processedgene0.8mRNA1/1-70
0905140A_eglinc/1-70
57 NPGTNVVNHVPHVG 70
57 NPGTNVVNHVPHVG 70
Conservation
Quality
Consensus
NPGTNVVNHVPHVG
Occupancy

B

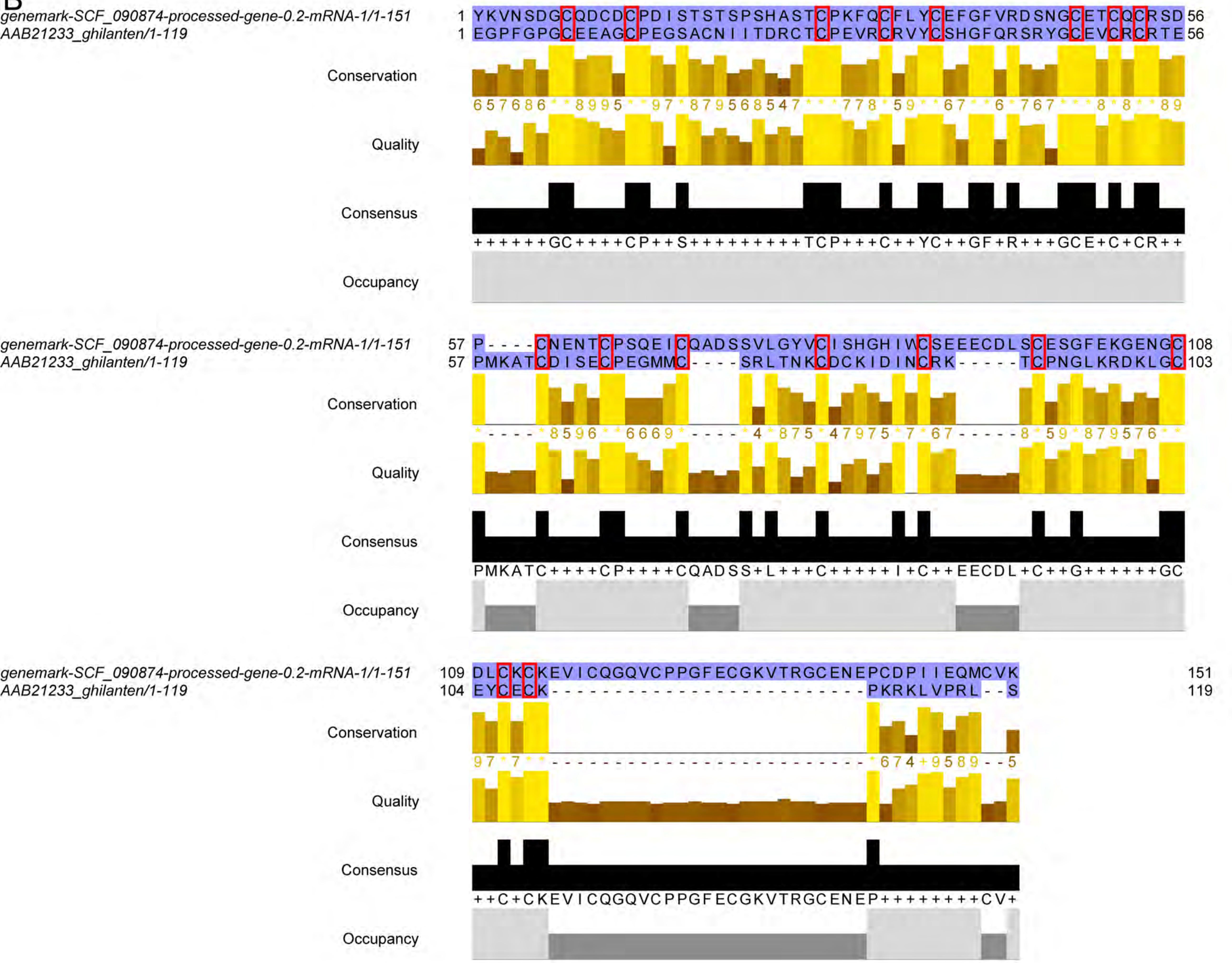
genemark-SCF_090874-processed-gene-0.2-mRNA-1/1-151
AAB21233_ghilanten/1-119
Conservation
Quality
Consensus
Occupancy

C

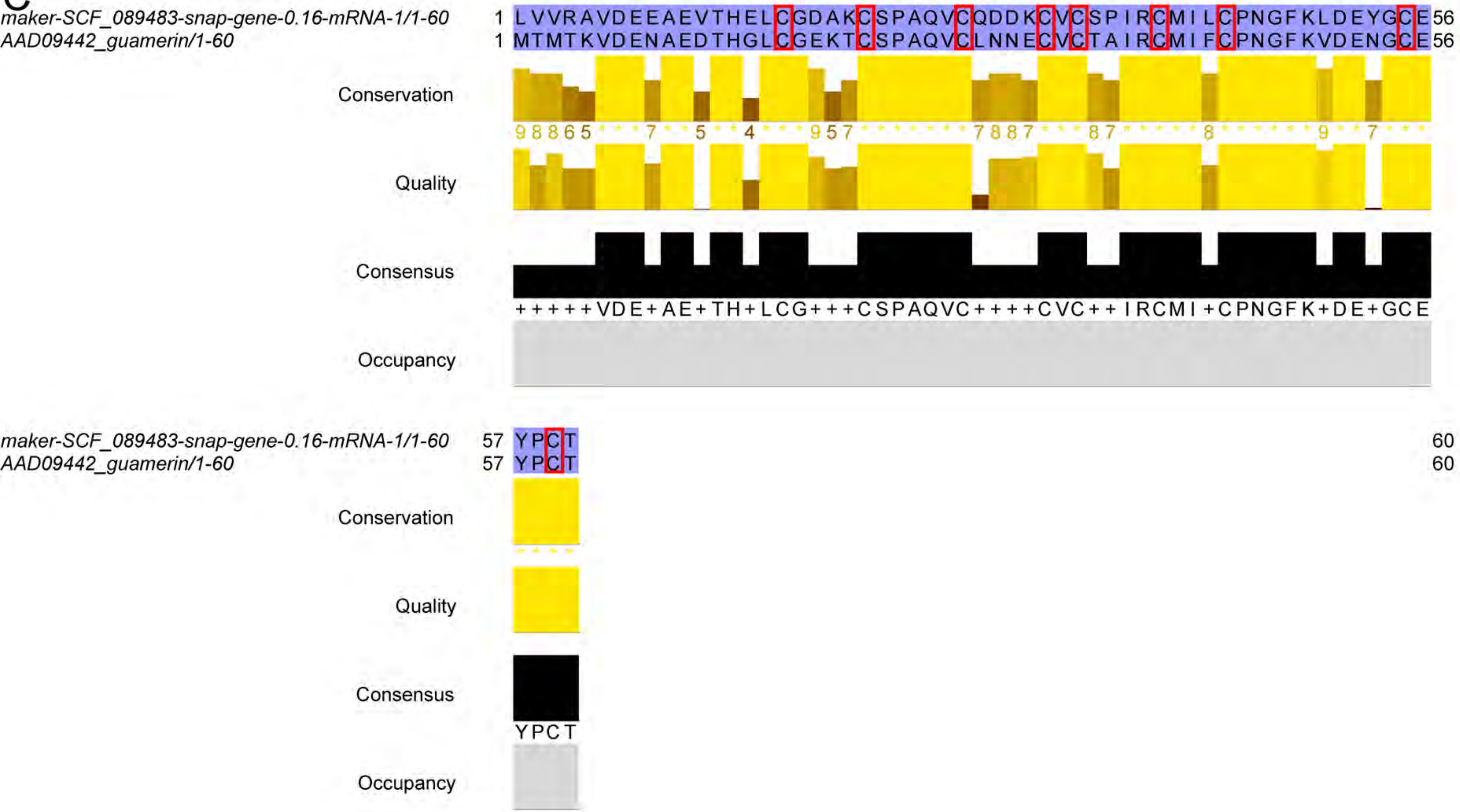

maker-SCF_089483-snap-gene-0.16-mRNA-1/1-60
AAD09442_guamerin/1-60
1 LVVRAVDEEAEVTHELCGDAKCSPAQVCQDDKCVCSPIRCMILCPNGFKLDEYGCE 56
1 MTMTKVDENAEDTHGLCGEKTCSPAQVCLNNECVCTAIRCMIFCPNGFKVDENGCE 56
Conservation
Quality
Consensus
+++++VDE+AE+TH+LCG+++CSPAQVC++++CVC++IRCMI+CPNGFK+DE+GCE
Occupancy
57 YPCT 60
57 YPCT 60
YPCT

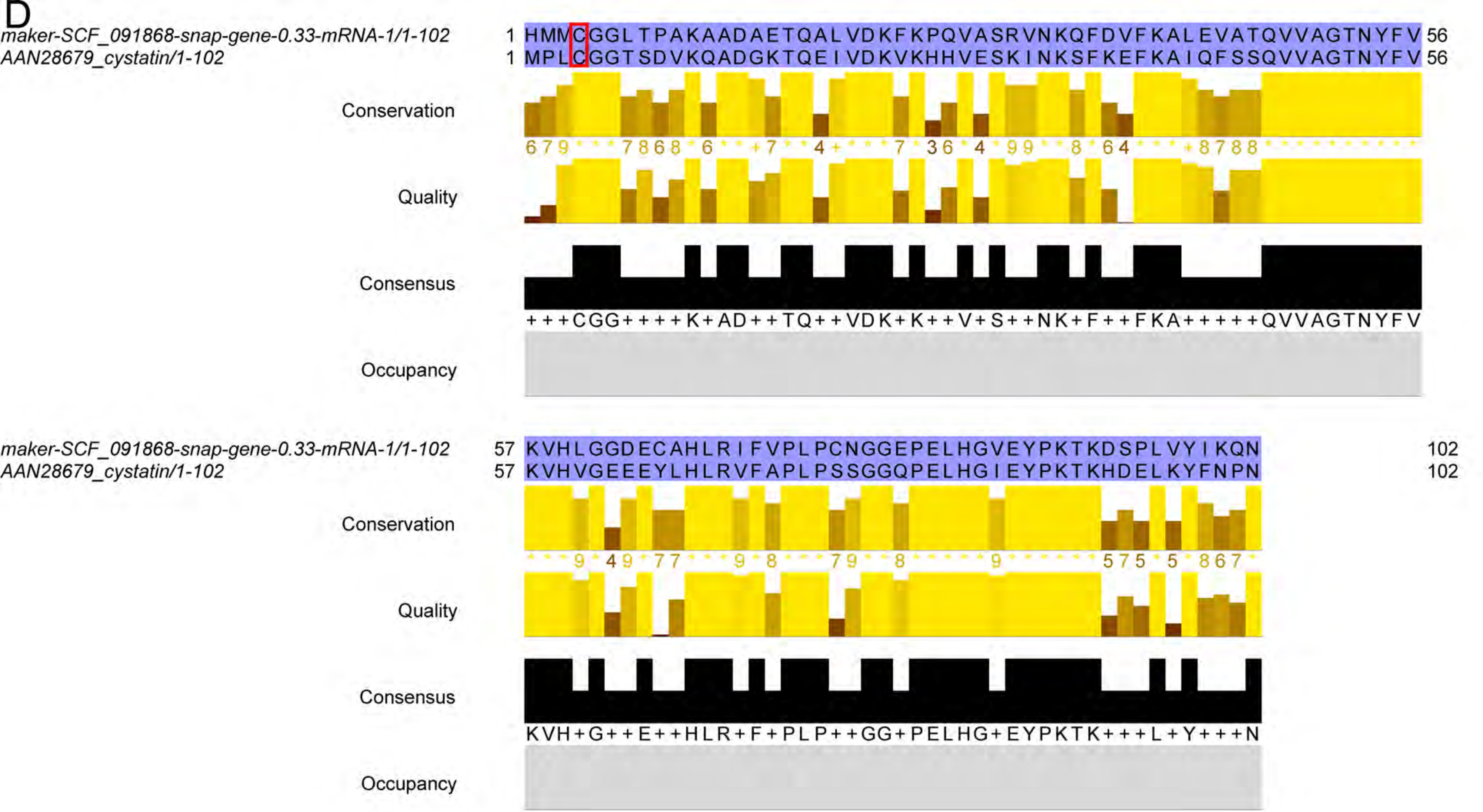
D
maker-SCF_091868-snap-gene-0.33-mRNA-1/1-102 1 HMMCGGLTPAKAADAETQALVDKFKPQVASRVNKQFDVFKALEVATQVVAGTNYFV 56
AAN28679_cystatin/1-102 1 MPLCGGTSDVKQADGKTQEIVDKVKHHVESKINKSFKEFKAIQFSSQVVAGTNYFV 56
Conservation
Quality
Consensus
+++CGG++++K+AD++TQ++VDK+K++V+S++NK+F++FKA+++++QVVAGTNYFV
Occupancy
maker-SCF_091868-snap-gene-0.33-mRNA-1/1-102 57 KVHLGGDECAHLRIFVPLPCNGGEPELHGVEYPKTKDSPLVYIKQN 102
AAN28679_cystatin/1-102 57 KVHVGEEEYLHLRVFAPLPSSGGQPELHGIEYPKTKHDELKYFNPN 102
Conservation
Quality
Consensus
KVH+G++E++HLR+F+PLP++GG+PELHG+EYPKTK+++L+Y+++N
Occupancy

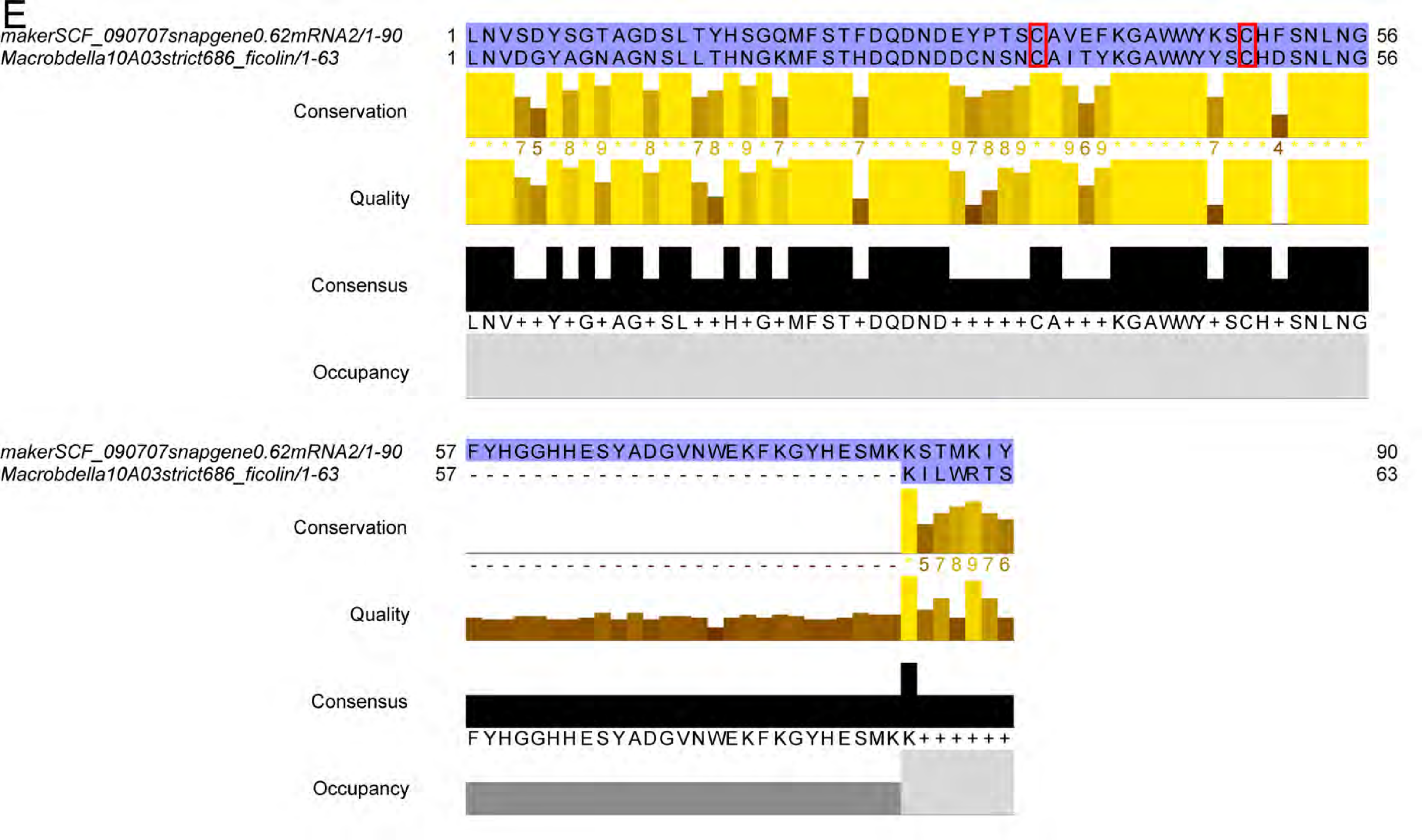
E
makerSCF_090707snapgene0.62mRNA2/1-90 1 LNVSDYSGTAGDSLTYHSGQMFSTFDQDNDEYPTSCAVEFKGAWWYKSCHFSNLNG 56
Macrobdella10A03strict686_ficolin/1-63 1 LNVDGYAGNAGNSLLTHNGKMFSTHDQDNDDCNSNCAITYKGAWWYSCHDSNLNG 56
Conservation
Quality
Consensus
LNV++Y+G+AG+SL++H+G+MFST+DQDND+++++CA+++KGAWWY+SCH+SNLNG
Occupancy
makerSCF_090707snapgene0.62mRNA2/1-90 57 FYHGGHHESYADGVNWEKFKGYHESMKKSTMKIY 90
Macrobdella10A03strict686_ficolin/1-63 57 ----------------------------KILWRTS 63
Conservation
Quality
Consensus
FYHGGHHESYADGVNWEKFKGYHESMKK++++++
Occupancy

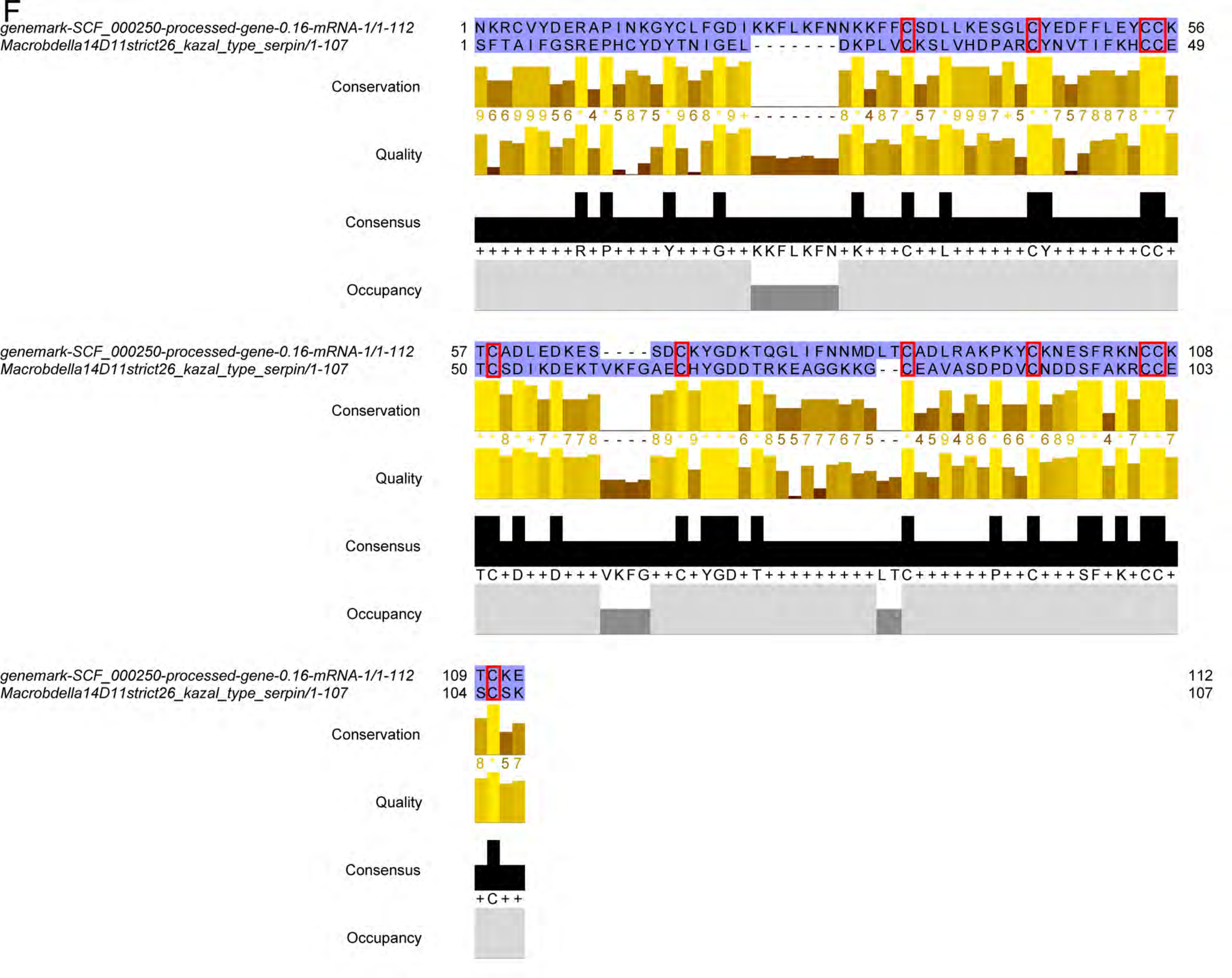
F
genemark-SCF_000250-processed-gene-0.16-mRNA-1/1-112 1 NKRCVYDERAPINKGYCLFGDIKKFLKFNNKKFFCSDLLKESGLCYEDFFLEYCCK 56
Macrobdella14D11strict26_kazal_type_serpin/1-107 1 SFTAIFGSREPHCYDYTNIGEL-------DKPLVCKSLVHDPARCYNVTIFKHCCE 49
Conservation
Quality
Consensus
++++++++R+P++++Y+++G++KKFLKFN+K+++C++L++++++CY+++++++CC+
Occupancy
genemark-SCF_000250-processed-gene-0.16-mRNA-1/1-112 57 TCADLEDKES----SDCKYGDKTQGLIFNNMDLTCADLRAKPKYCKNESFRKNCCK 108
Macrobdella14D11strict26_kazal_type_serpin/1-107 50 TCSDIKDEKTVKFGAECHYGDDTRKEAGGKKG--CEAVASDPDVCNDDSFAKRCCE 103
Conservation
Quality
Consensus
TC+D++D+++VKFG++C+YGD+T+++++++++LTC++++++P++C+++SF+K+CC+
Occupancy
genemark-SCF_000250-processed-gene-0.16-mRNA-1/1-112 109 TCKE 112
Macrobdella14D11strict26_kazal_type_serpin/1-107 104 SCSK 107
Conservation
Quality
Consensus
+C++
Occupancy

G

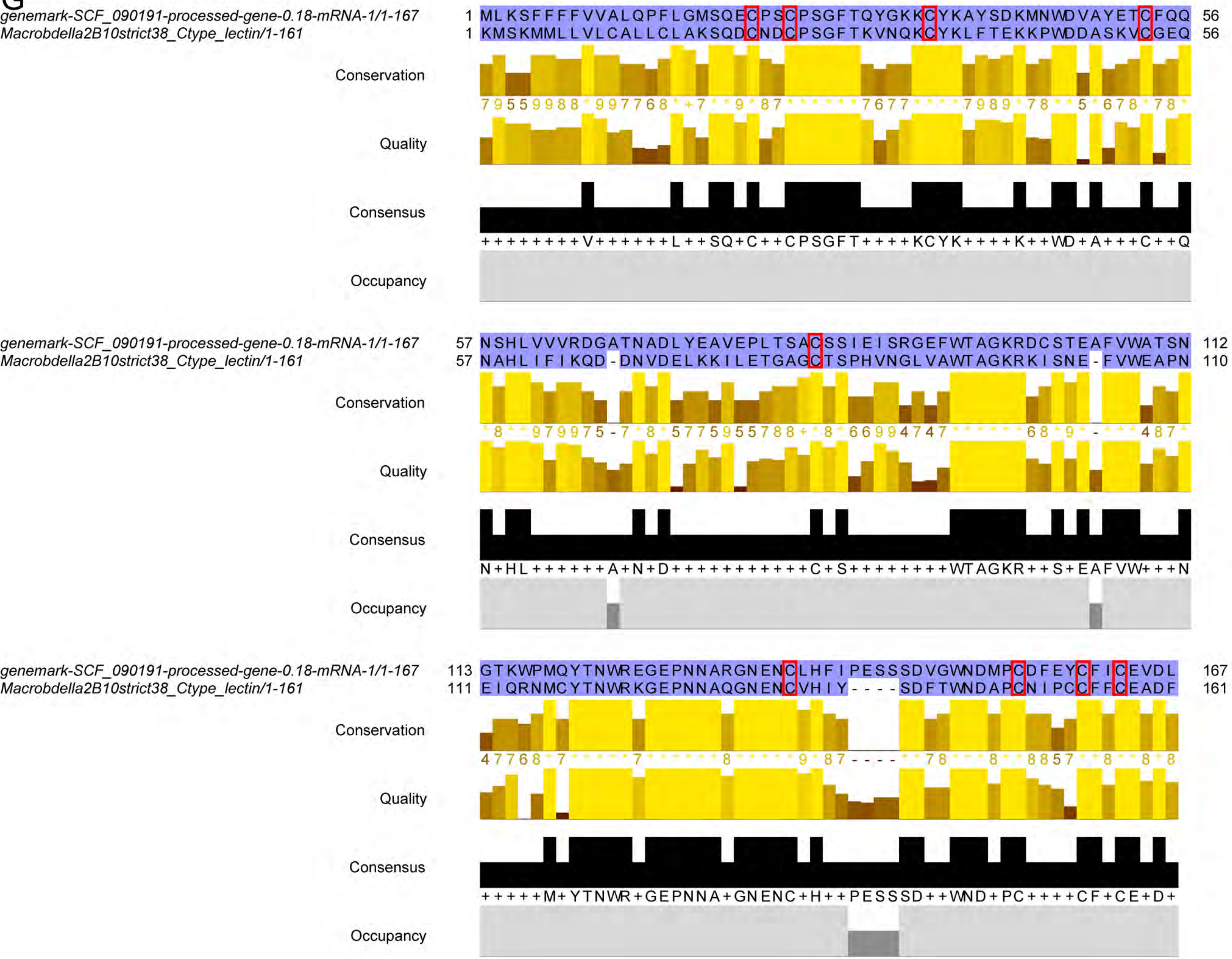

genemark-SCF_090191-processed-gene-0.18-mRNA-1/1-167
Macrobdella2B10strict38_Ctype_lectin/1-161
Conservation
Quality
Consensus
Occupancy

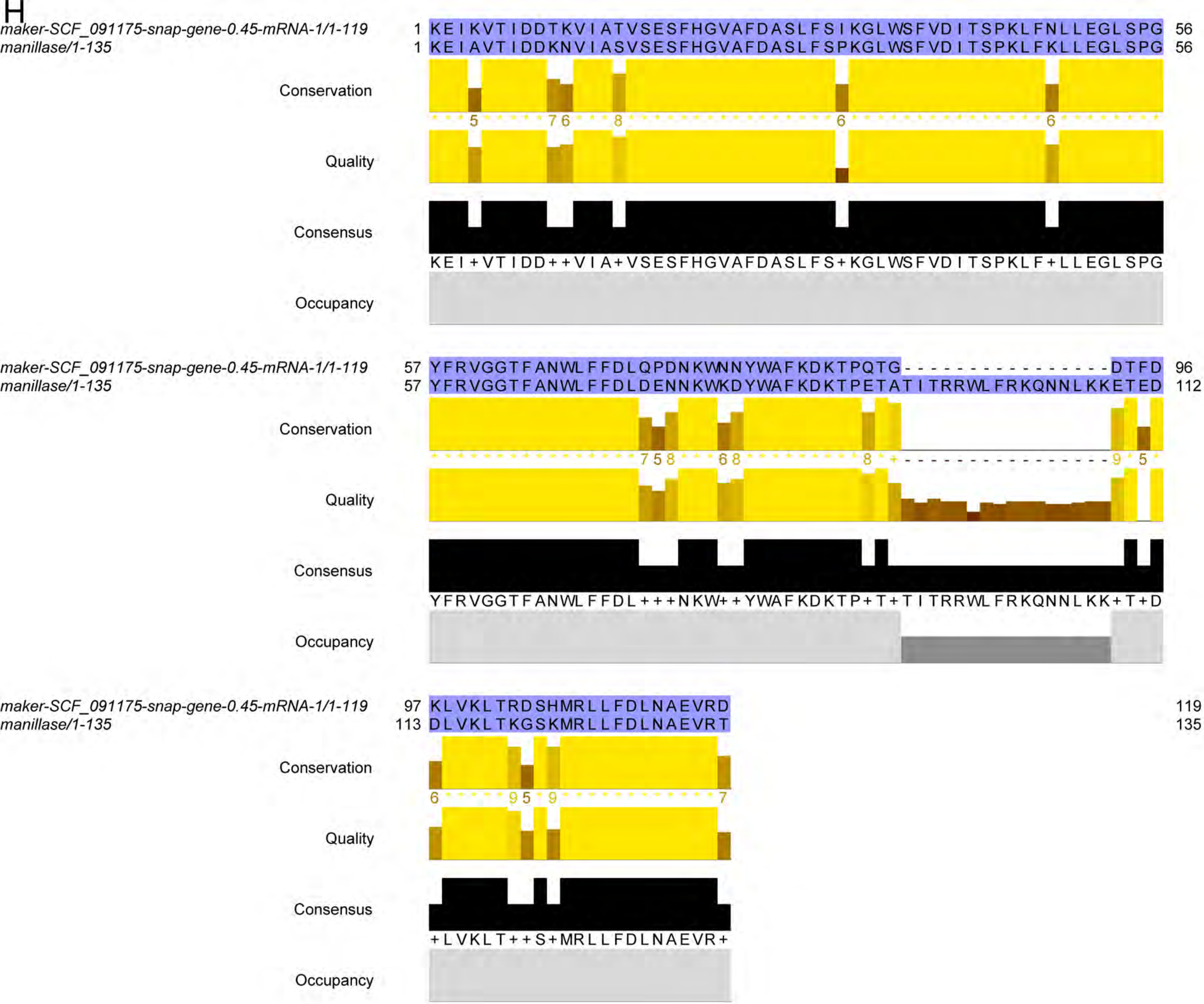
H
maker-SCF_091175-snap-gene-0.45-mRNA-1/1-119 1 KEIKVTIDDTKVIATVSESFHGVAFDASLFSIKGLWSFVDITSPKLFNLLEGLSPG 56
manillase/1-135 1 KEIAVTIDDKNVIASVSESFHGVAFDASLFSPKGLWSFVDITSPKLFKLLEGLSPG 56
Conservation
Quality
Consensus
KEI+VTIDD++VIA+VSESFHGVAFDASLFS+KGLWSFVDITSPKLF+LLEGLSPG
Occupancy
maker-SCF_091175-snap-gene-0.45-mRNA-1/1-119 57 YFRVGGTFANWLFFDLQPDNKWNNYWAFKDKTPQTG----------------DTFD 96
manillase/1-135 57 YFRVGGTFANWLFFDLDENNKWKDYWAFKDKTPETATITRRWLFRKQNNLKKETED 112
Conservation
Quality
Consensus
YFRVGGTFANWLFFDL+++NKW++YWAFKDKTP+T+TITRRWLFRKQNNLKK+T+D
Occupancy
maker-SCF_091175-snap-gene-0.45-mRNA-1/1-119 97 KLVKLTRDSHMRLLFDLNAEVRD 119
manillase/1-135 113 DLVKLTKGSKMRLLFDLNAEVRT 135
Conservation
Quality
Consensus
+LVKLT++S+MRLLFDLNAEVR+
Occupancy

I

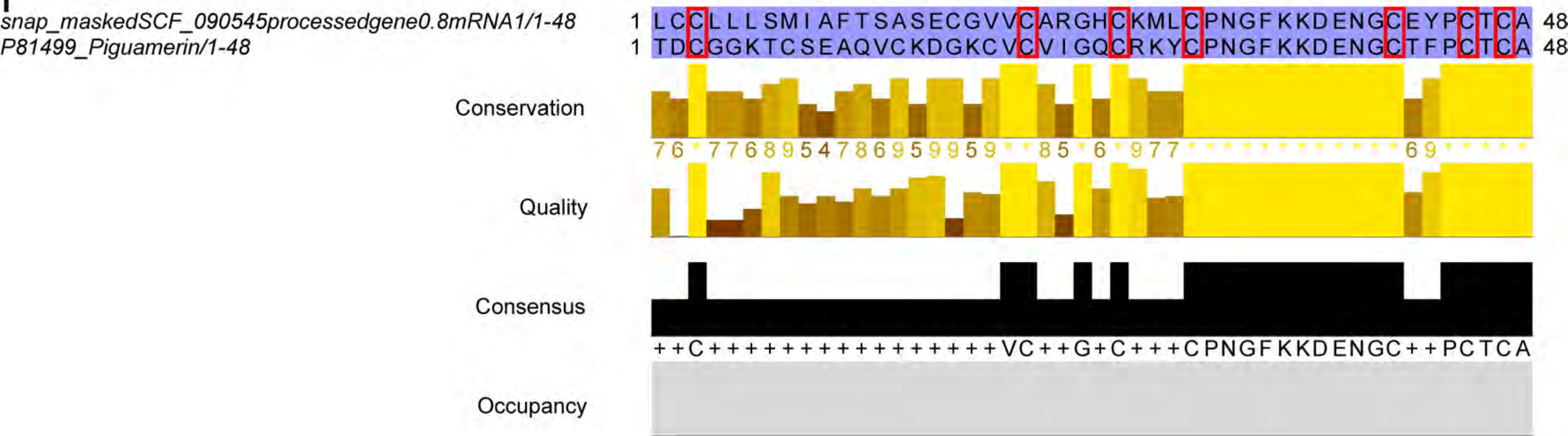
snap_maskedSCF_090545processedgene0.8mRNA1/1-48
1 LCCLLLSMIAFTSASECGVVCARGHCKMLCPNGFKKDENGCEYPCTCA 48
P81499_Piguamerin/1-48
1 TDCGGKTCSEAQVCKDGKCVCVIGQCRKYCPNGFKKDENGCTFPCTCA 48
Conservation
76*776895478695995 9**85*6*977************69*****
Quality
Consensus
++C+++++++++++++++++VC++G+C+++CPNGFKKDENGC++PCTCA
Occupancy

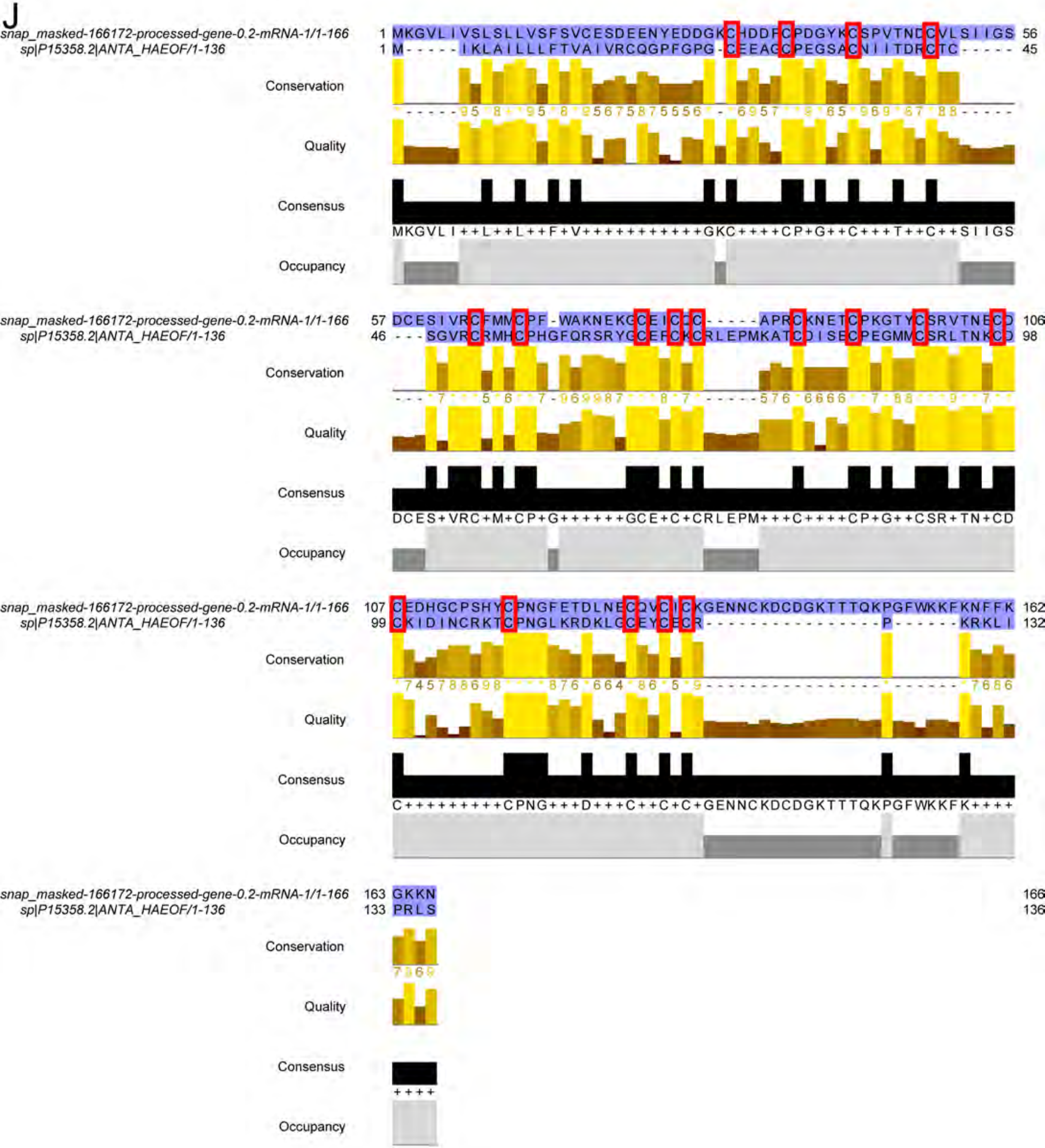
J
snap_masked-166172-processed-gene-0.2-mRNA-1/1-166
sp|P15358.2|ANTA_HAEOF/1-136
Conservation
Quality
Consensus
Occupancy

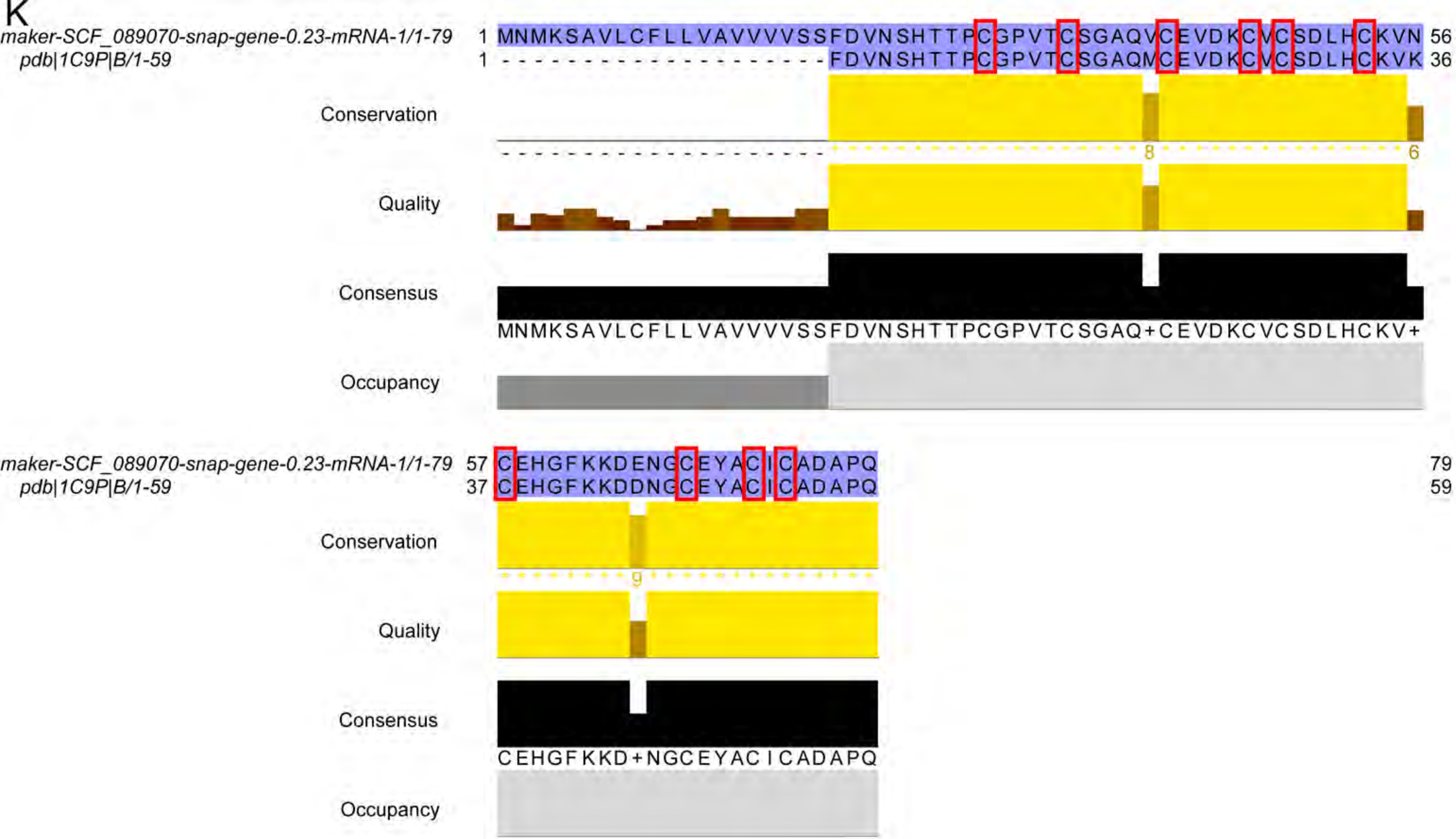
K
maker-SCF_089070-snap-gene-0.23-mRNA-1/1-79 1 MNMKSAVLCFLLVAVVVVSSFDVNSHTTPCGPVTCSGAQVCEVDKCVCSDLHCKVN 56
pdb|1C9P|B/1-59 1 --------------------FDVNSHTTPCGPVTCSGAQMCEVDKCVCSDLHCKVK 36
Conservation
8
6
Quality
Consensus
MNMKSAVLCFLLVAVVVVSSFDVNSHTTPCGPVTCSGAQ+CEVDKCVCSDLHCKV+
Occupancy
maker-SCF_089070-snap-gene-0.23-mRNA-1/1-79 57 CEHGFKKDENGCEYACICADAPQ 79
pdb|1C9P|B/1-59 37 CEHGFKKDDNGCEYACICADAPQ 59
Conservation
9
Quality
Consensus
CEHGFKKD+NGCEYACICADAPQ
Occupancy

L

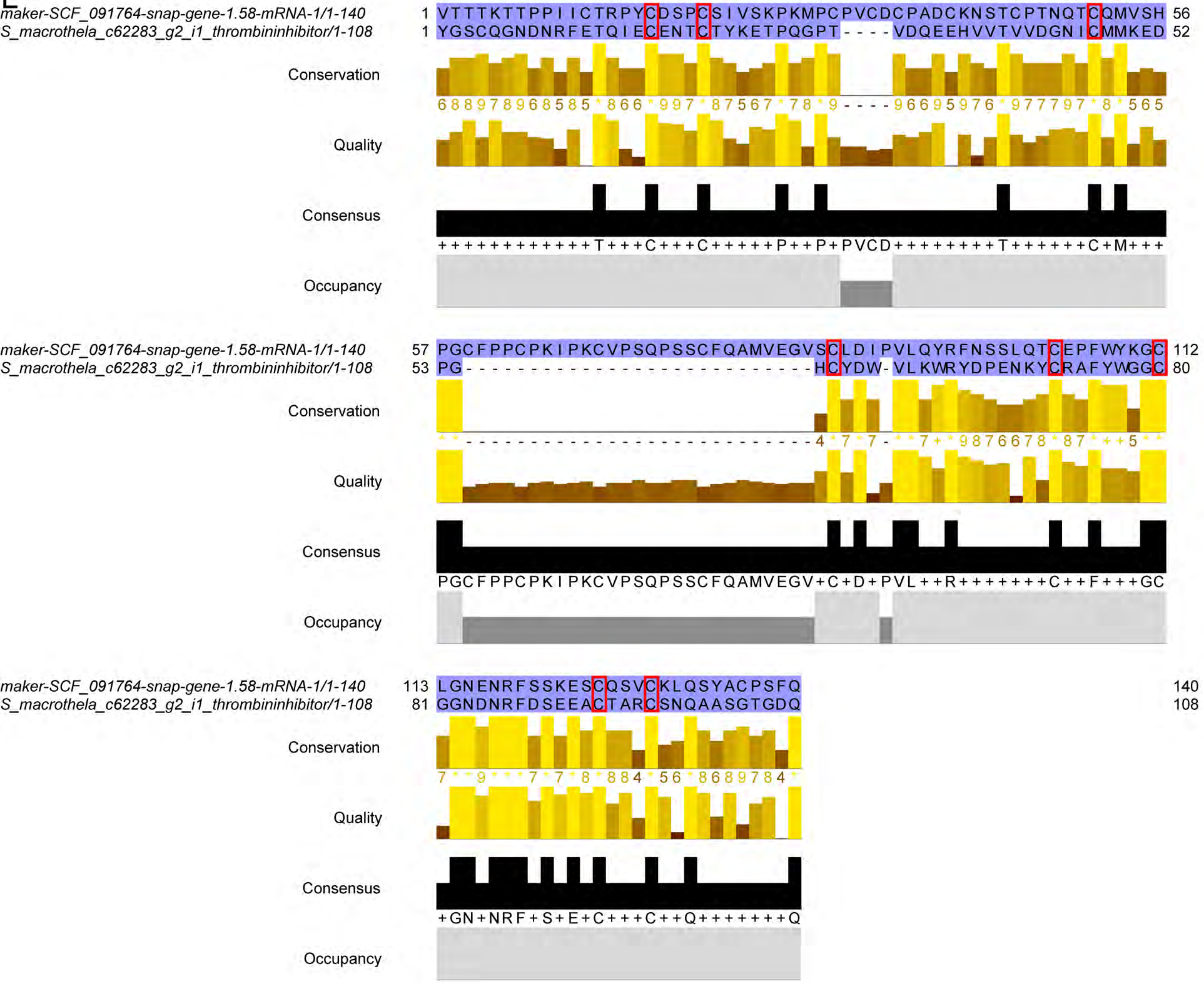
maker-SCF_091764-snap-gene-1.58-mRNA-1/1-140 1 VTTTKTTPPIICTRPYCDSPCSIVSKPKMPCPVCDCPADCKNSTCPTNQTCQMVSH 56
S_macrothela_c62283_g2_i1_thrombininhibitor/1-108 1 YGSCQGNDNRFETQIECENTCTYKETPQGPT----VDQEEHVVTVVDGNICMMKED 52
Conservation
Quality
Consensus
++++++++++++T+++C+++C+++++P++P+PVCD++++++++T++++++C+M+++
Occupancy
maker-SCF_091764-snap-gene-1.58-mRNA-1/1-140 57 PGCFPPCPKIPKCVPSQPSSCFQAMVEGVSCLDIPVLQYRFNSSLQTCEPFWYKGC 112
S_macrothela_c62283_g2_i1_thrombininhibitor/1-108 53 PG---------------------------HCYDW-VLKWRYDPENKYCRAFYWGGC 80
Conservation
Quality
Consensus
PGCFPPCPKIPKCVPSQPSSCFQAMVEGV+C+D+PVL++R++++++++C++F+++GC
Occupancy
maker-SCF_091764-snap-gene-1.58-mRNA-1/1-140 113 LGNENRFSSKESCQSVCKLQSYACPSFQ 140
S_macrothela_c62283_g2_i1_thrombininhibitor/1-108 81 GGNDNRFDSEEACTARCSNQAASGTGDQ 108
Conservation
Quality
Consensus
+GN+NRF+S+E+C+++C++Q+++++++Q
Occupancy
