# Supplementary material for: Draft genome of the European medicinal leech Hirudo medicinalis (Annelida, Clitellata, Hirudiniformes) with emphasis on anticoagulants
Source: Sci Rep. 2020 Jun 18;10:9885. doi: 10.1038/s41598-020-66749-5 (PMC7303139; doi:10.1038/s41598-020-66749-5)
Supplement: Supplementary file 4 — Supplementary Information 4. [file 41598_2020_66749_MOESM4_ESM.pdf]

A

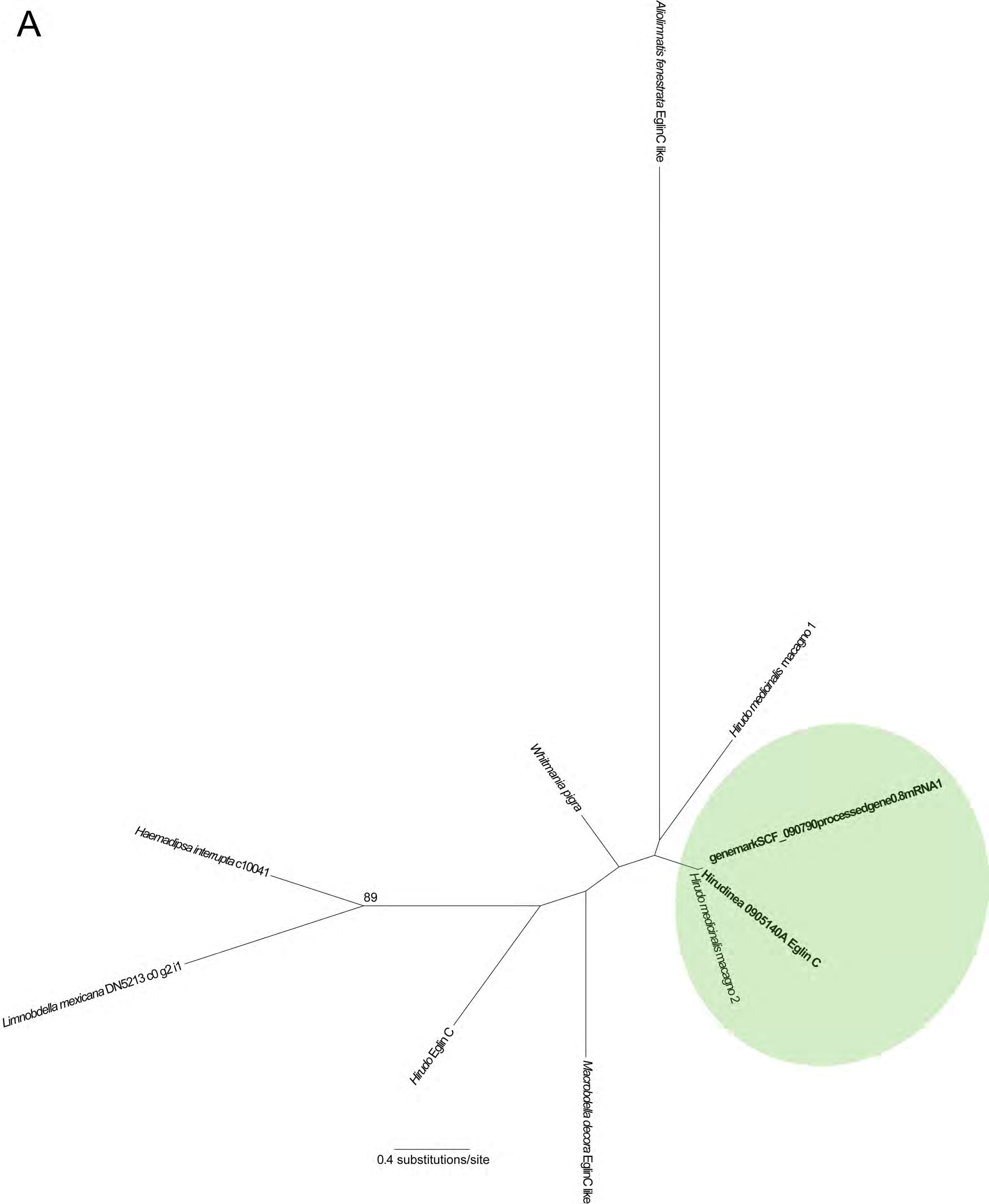

Alilimnatis fenestrata EglinC like
Hirudo medicinalis macagno 1
Whitmania pigra
genemarkSCF_090790processedgene0.8mRNA1
Haemadipsa interrupta c10041
Hirudinea 0905140A Eglin C
Hirudo medicinalis macagno 2
89
Limnobdella mexicana DN5213 c0 g2 i1
Hirudo Eglin C
Macrobdella decora EglinC like
0.4 substitutions/site

Draft genome of the European medicinal leech *Hirudo medicinalis* (Annelida, Clitellata, Hirudiniformes) with emphasis on anticoagulants

Sebastian Kvist[1,2,*], Alejandro Manzano-Marín[3], Danielle de Carle[1,2], Peter Trontelj[4] & Mark E. Siddall[5]

[1] Department of Natural History, Royal Ontario Museum, 100 Queen's Park, Toronto, ON M5S 2C6, Canada

[2] Department of Ecology and Evolutionary Biology, University of Toronto, 25 Willcocks Street, Toronto, ON M5S 2B4, Canada

[3] Centre for Microbiology and Environmental Systems Science, University of Vienna, 1090 Vienna, Austria

[4] Department of Biology, Biotechnical Faculty, University of Ljubljana, Jamnikarjeva 101, 1000 Ljubljana, Slovenia

[5] Division of invertebrate Zoology, American Museum of Natural History, 79th Street @ Central Park West, New York, NY 10025, USA.

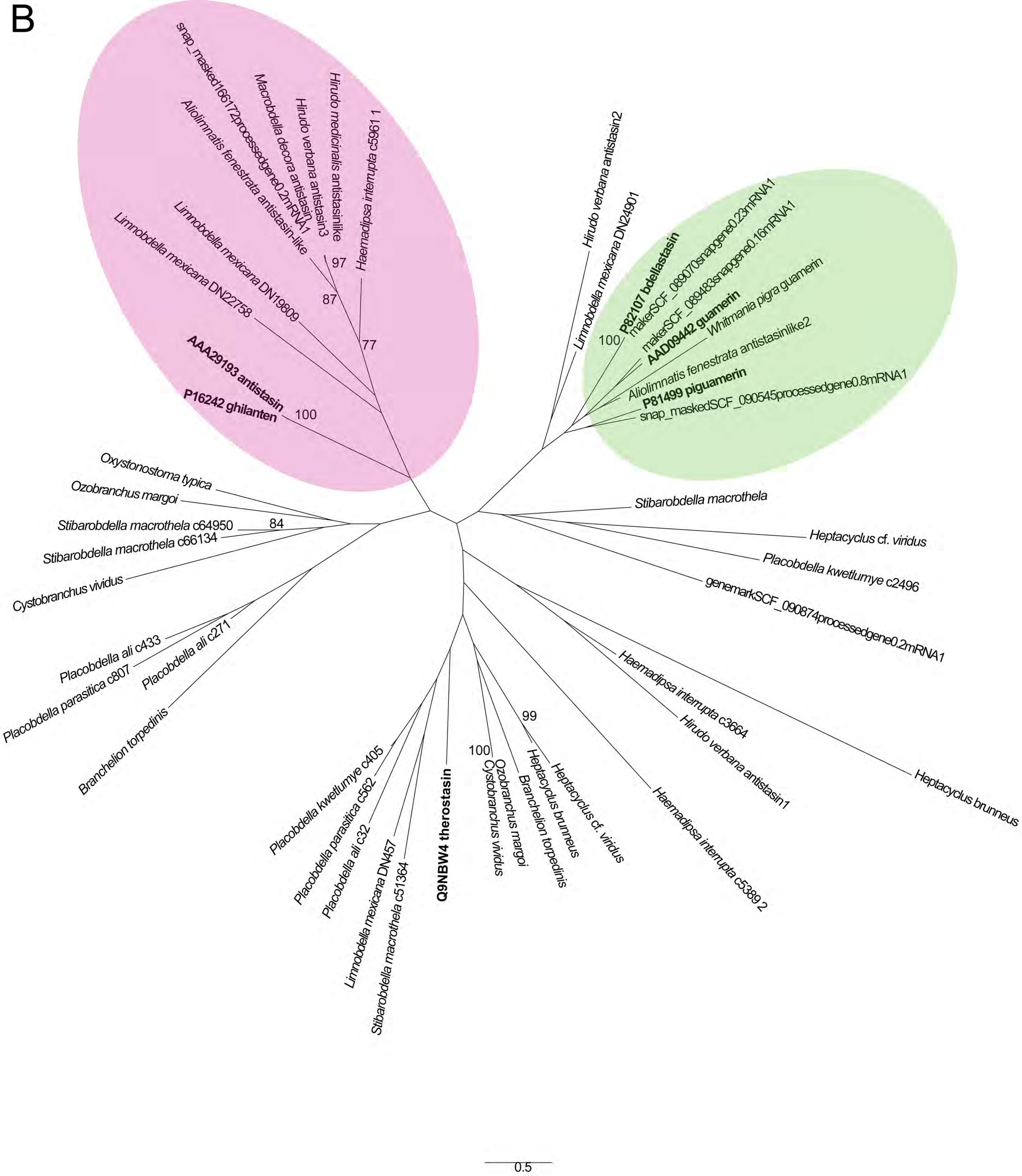
B
snap_masked166172processedgene0.2mRNA1
Macrobdella decora antistasin3
Hirudo verbana antistasin
Hirudo medicinalis antistasinlike
Haemadipsa interrupta c5961 1
Aliolimnatis fenestrata antistasin-like
Limnobdella mexicana DN19809
Limnobdella mexicana DN22758
AAA29193 antistasin
P16242 ghilanten
97
87
77
100
Hirudo verbana antistasin2
Limnobdella mexicana DN24901
P82107 bdellastasin
makerSCF_089070snapgene0.23mRNA1
makerSCF_089483snapgene0.16mRNA1
AAD09442 guamerin
Whitmania pigra guamerin
Aliolimnatis fenestrata antistasinlike2
P81499 piguamerin
snap_maskedSCF_090545processedgene0.8mRNA1
100
Oxystonostoma typica
Ozobranchus margoi
Stibarobdella macrothela c64950
Stibarobdella macrothela c66134
84
Cystobranchus vividus
Stibarobdella macrothela
Heptacyclus cf. viridus
Placobdella kwetlumye c2496
genemarkSCF_090874processedgene0.2mRNA1
Placobdella ali c433
Placobdella parasitica c807
Placobdella ali c271
Branchelion torpedinis
Haemadipsa interrupta c3664
Hirudo verbana antistasin1
Heptacyclus brunneus
Haemadipsa interrupta c5389 2
99
Heptacyclus cf. viridus
Heptacyclus brunneus
Branchelion torpedinis
Ozobranchus margoi
Cystobranchus vividus
100
Q9NBW4 therostasin
Stibarobdella macrothela c51364
Limnobdella mexicana DN457
Placobdella ali c32
Placobdella parasitica c562
Placobdella kwetlumye c405
0.5

C

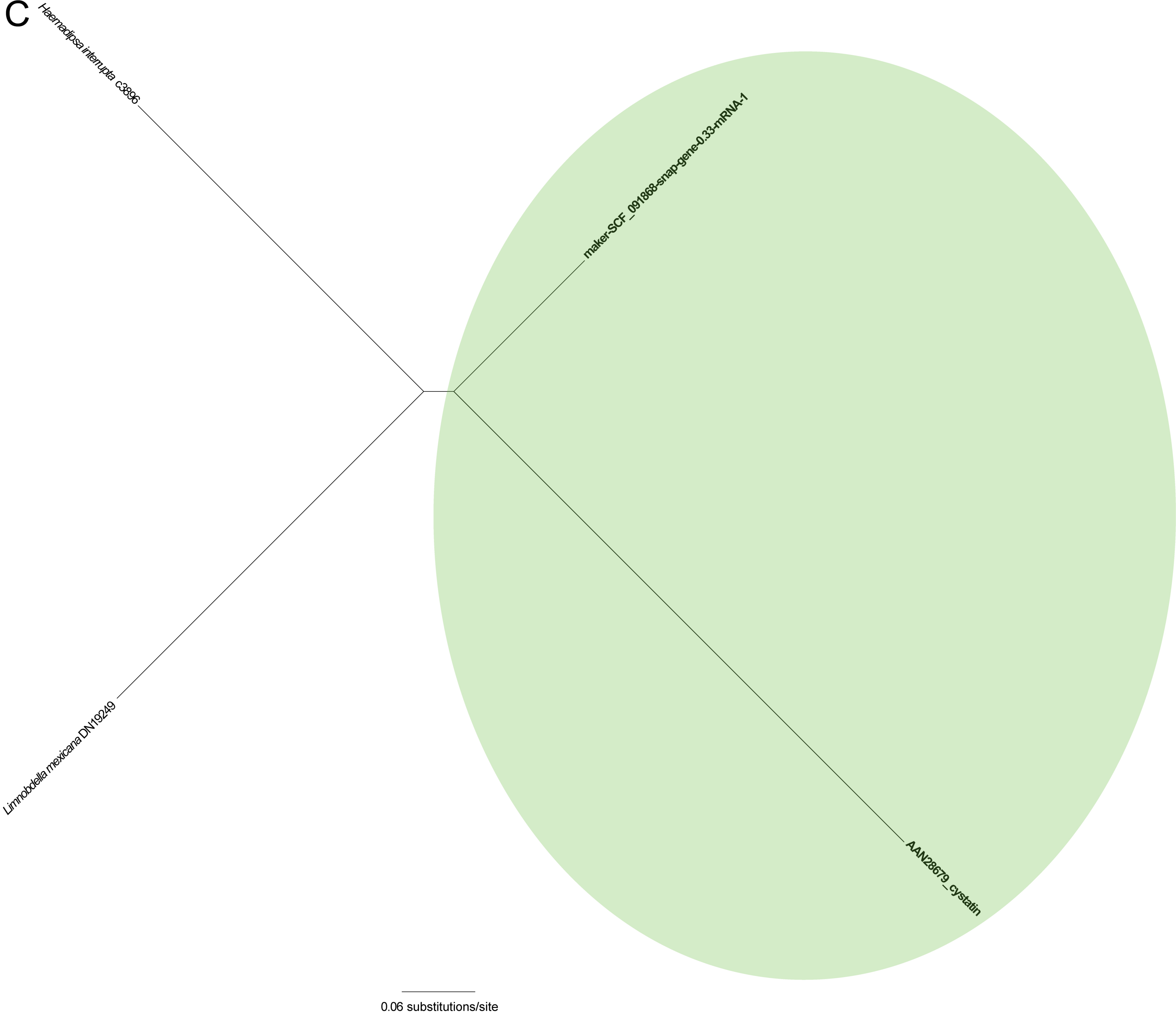
Haemadipsa interrupta c3896
maker-SCF_091868-snap-gene-0.33-mRNA-1
Limnobdella mexicana DN19249
AAN28679_cystatin
0.06 substitutions/site

D

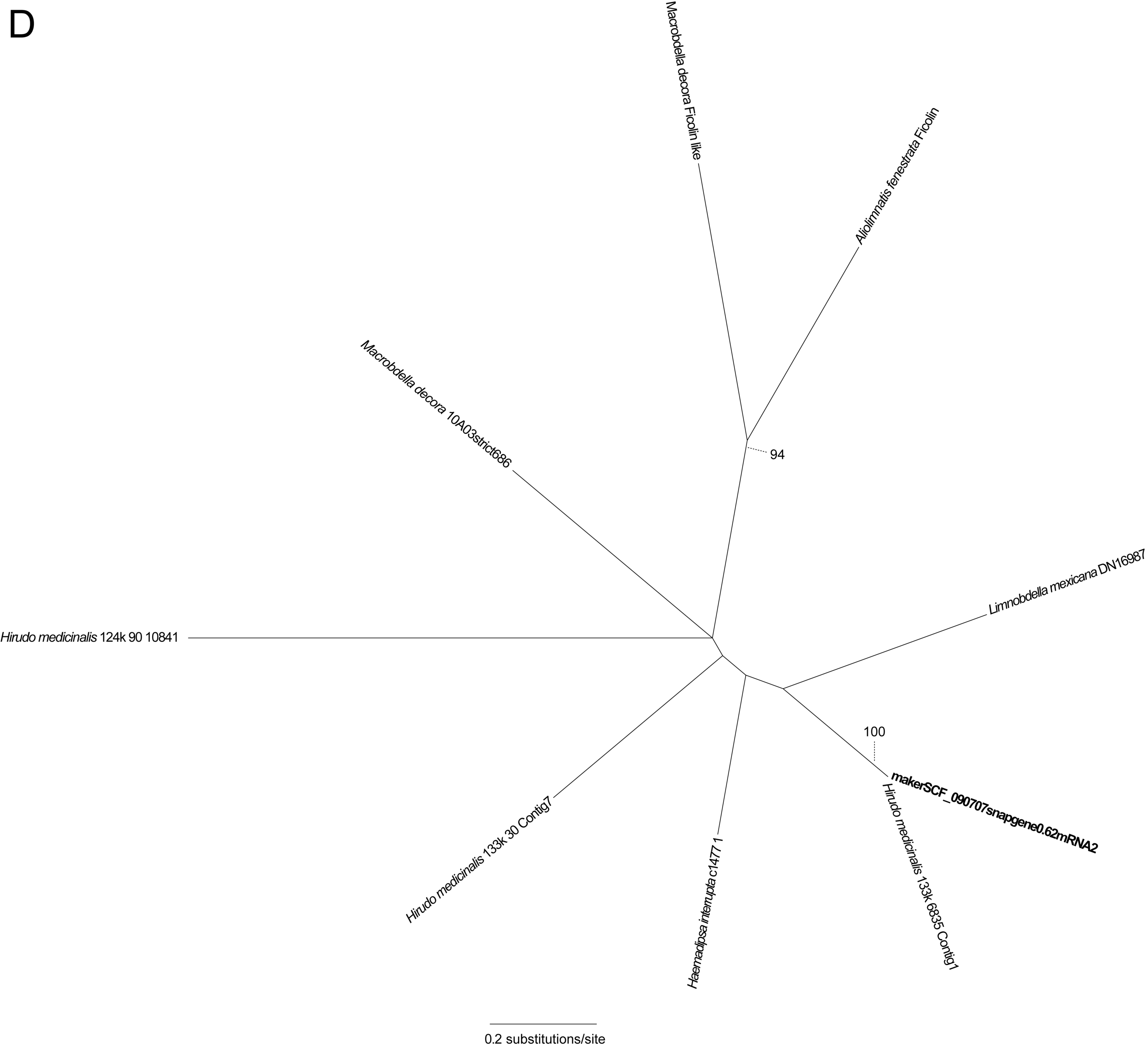
Macrobdella decora Ficolin like
Aliolimnatis fenestrata Ficolin
Macrobdella decora 10A03strict686
94
Limnobdella mexicana DN16987
Hirudo medicinalis 124k 90 10841
100
makerSCF_090707snapgene0.62mRNA2
Hirudo medicinalis 133k 6835 Contig1
Haemadipsa interrupta c1477 1
Hirudo medicinalis 133k 30 Contig7
0.2 substitutions/site

E

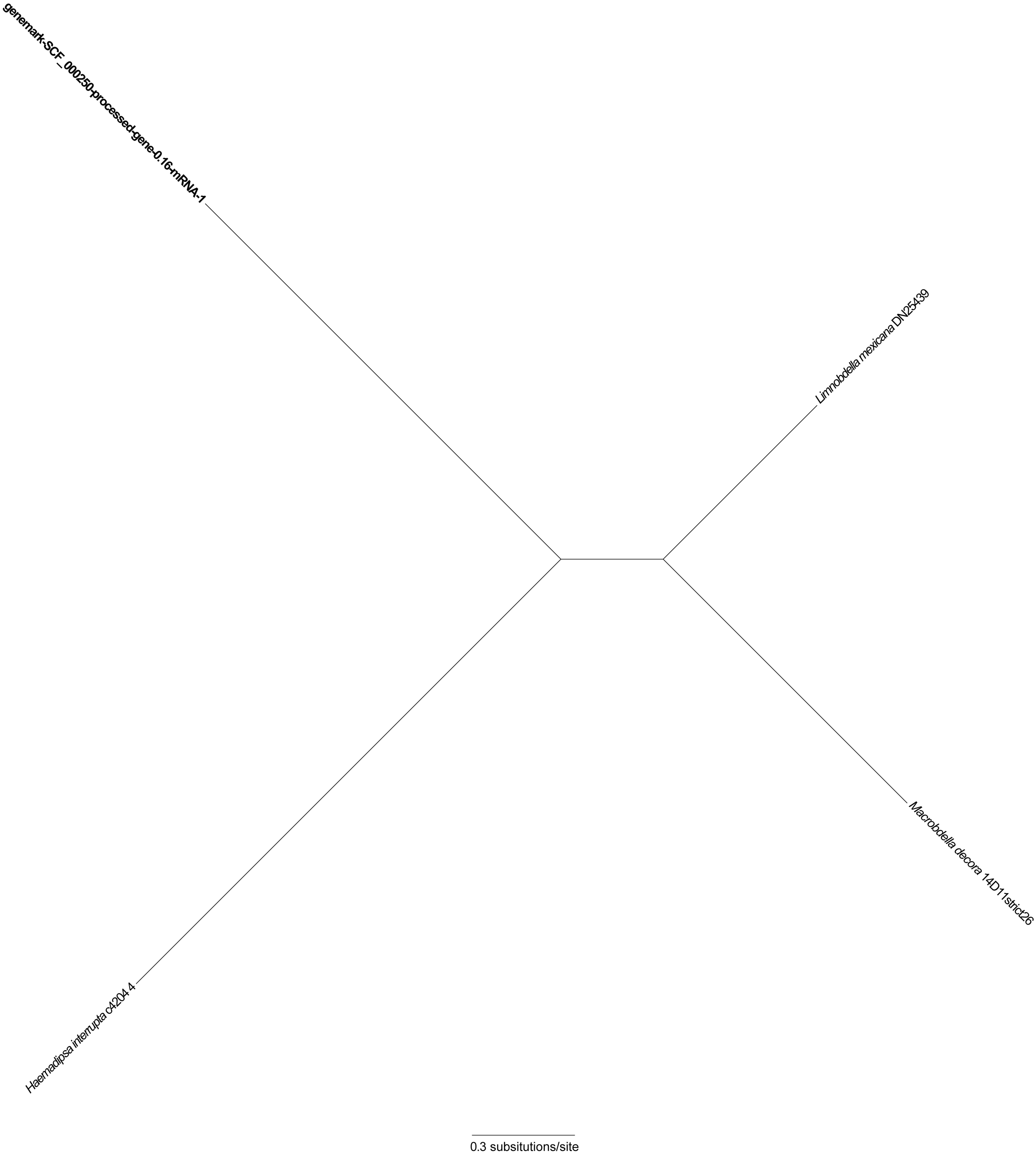
genemark-SCF_000250-processed-gene-0.16-mRNA-1
Limnobdella mexicana DN25439
Macrobdella decora 14D11strict26
Haemadipsa interrupta c4204.4
0.3 subsitutions/site

F

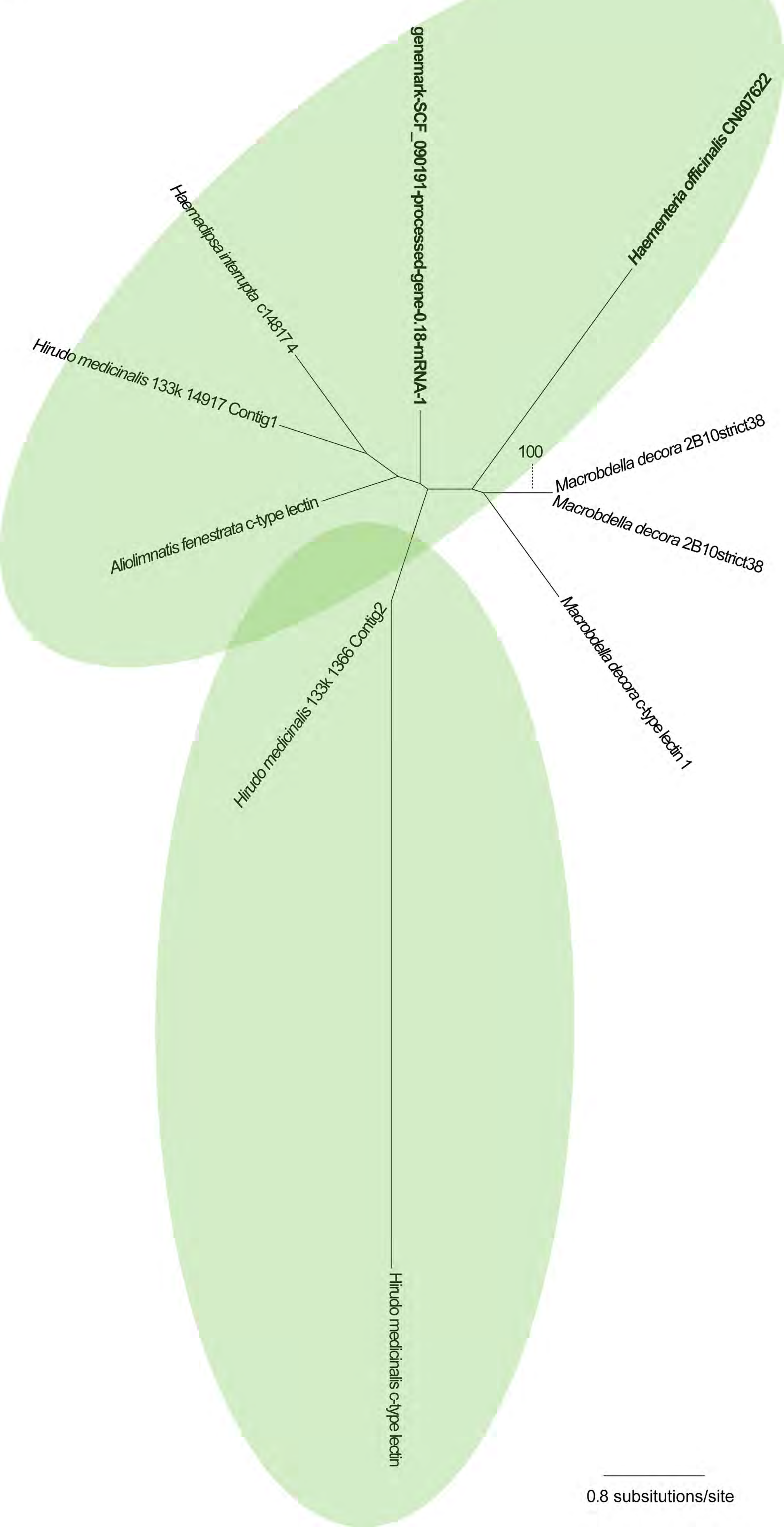
genemark-SCF_090191-processed-gene-0.18-mRNA-1
Haementeria officinalis CN807622
Haemadipsa interrupta c148174
Hirudo medicinalis 133k 14917 Contig1
100
Macrobdella decora 2B10strict38
Macrobdella decora 2B10strict38
Aliolimnatis fenestrata c-type lectin
Macrobdella decora c-type lectin 1
Hirudo medicinalis 133k 1366 Contig2
Hirudo medicinalis c-type lectin
0.8 subsitutions/site

G

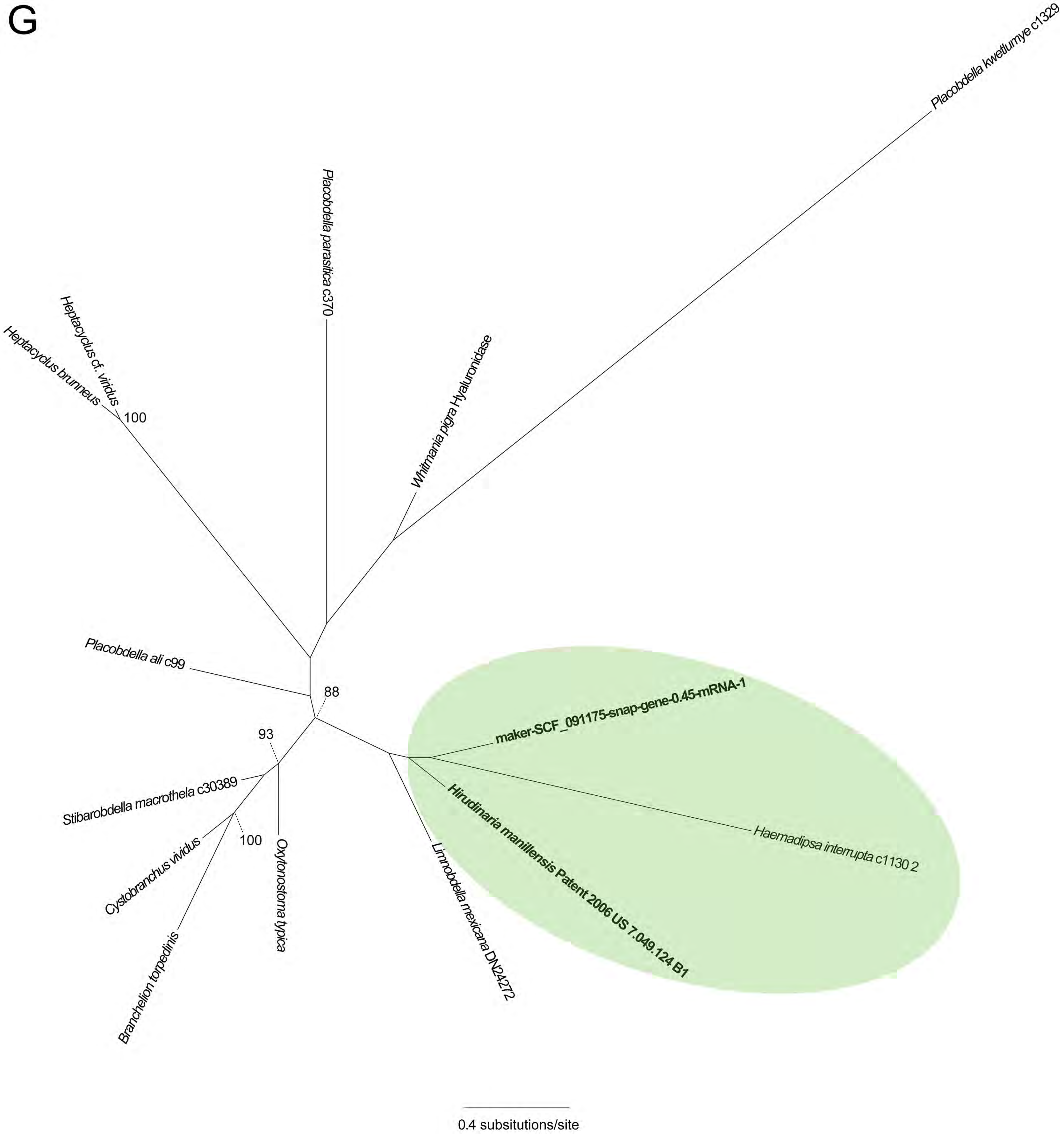

Placobdella kwetlumye c1329
Placobdella parasitica c370
Heptacyclus cf. viridus
Heptacyclus brunneus
100
Whitmania pigra Hyaluronidase
Placobdella ali c99
88
maker-SCF_091175-snap-gene-0.45-mRNA-1
93
Stibarobdella macrothela c30389
Hirudinaria manillensis Patent 2006 US 7.049.124 B1
Haemadipsa interrupta c1130 2
Cystobranchus vividus
100
Oxytonostoma typica
Limnobdella mexicana DN24272
Branchelion torpedinis
0.4 subsitutions/site
